# Exploring Off-Targets and Off-Systems for Adverse Drug Reactions via Chemical-Protein Interactome — Clozapine-Induced Agranulocytosis as a Case Study

**DOI:** 10.1371/journal.pcbi.1002016

**Published:** 2011-03-31

**Authors:** Lun Yang, Kejian Wang, Jian Chen, Anil G. Jegga, Heng Luo, Leming Shi, Chunling Wan, Xizhi Guo, Shengying Qin, Guang He, Guoyin Feng, Lin He

**Affiliations:** 1 Bio-X Center, Key Laboratory for the Genetics of Developmental and Neuropsychiatric Disorders (Ministry of Education), Shanghai Jiao Tong University, Shanghai, China; 2 Institutes of Biomedical Sciences, Fudan University, Shanghai, China; 3 School of Pharmacy, Fudan University, Shanghai, China; 4 Division of Biomedical Informatics, Cincinnati Children's Hospital Medical Center, Cincinnati, Ohio, United States of America; 5 Departments of Pediatrics and Computer Science, University of Cincinnati, Cincinnati, Ohio, United States of America; 6 Institute for Nutritional Sciences, Shanghai Institute of Biological Sciences, Chinese Academy of Sciences, Shanghai, China; Stanford University, United States of America

## Abstract

In the era of personalized medical practice, understanding the genetic basis of patient-specific adverse drug reaction (ADR) is a major challenge. Clozapine provides effective treatments for schizophrenia but its usage is limited because of life-threatening agranulocytosis. A recent high impact study showed the necessity of moving clozapine to a first line drug, thus identifying the biomarkers for drug-induced agranulocytosis has become important. Here we report a methodology termed as antithesis chemical-protein interactome (CPI), which utilizes the docking method to mimic the differences in the drug-protein interactions across a panel of human proteins. Using this method, we identified *HSPA1A*, a known susceptibility gene for CIA, to be the off-target of clozapine. Furthermore, the mRNA expression of *HSPA1A*-related genes (off-target associated systems) was also found to be differentially expressed in clozapine treated leukemia cell line. Apart from identifying the CIA causal genes we identified several novel candidate genes which could be responsible for agranulocytosis. Proteins related to reactive oxygen clearance system, such as oxidoreductases and glutathione metabolite enzymes, were significantly enriched in the antithesis CPI. This methodology conducted a multi-dimensional analysis of drugs' perturbation to the biological system, investigating both the off-targets and the associated off-systems to explore the molecular basis of an adverse event or the new uses for old drugs.

## Author Summary

Idiosyncratic drug reactions (IDR) generally cannot be identified until after a drug is taken by a large population, but usually result in restricted use or withdrawal. Clozapine provides the most effective treatment for schizophrenia but its use is limited because of a life-threatening IDR, i.e., the agranulocytosis. A high impact clinical study demonstrated the necessity of moving clozapine from 3^rd^ line to 1^st^ line drug; therefore, intensive research has aimed at identifying genes responsible for clozapine-induced agranulocytosis (CIA). Olanzapine, an analog of clozapine, has much lower incidence of agranulocytosis. Based on this phenomenon, we proposed an *in silico* methodology termed as antithesis chemical-protein interactome (CPI), which mimics the differences in the drug-protein interactions of the two drugs across a panel of human proteins. e.g., *HSPA1A* was identified to be targeted by clozapine not olanzapine. Furthermore, the gene expression of the *HSPA1A*-related gene system was also found up-regulated after clozapine treatment. This approach can examine the system's perturbation in terms of both the off-target and the off-system's interaction with the drug, providing theoretical basis for decoding the adverse drug reactions or the new uses for old drugs.

## Introduction

Clozapine (CLZ) provides one of the most effective therapeutic treatments for schizophrenia [1]. It is classified as an atypical antipsychotic drug because of its binding to serotonergic and dopamine receptors. However, its usage is limited due to potential life-threatening adverse drug reaction, mainly agranulocytosis [2], [3], [4]. FDA therefore requires blood testing for patients taking CLZ, complicating the clinical use of the drug. A recent high impact clinical study demonstrated the necessity of moving CLZ from a 3^rd^ line drug to a 1^st^ line drug based on its overall benefit/risk ratio [1]. Thus the identification of the biomarkers for clozapine induced agranulocytosis (CIA) could greatly broaden the usage of this drug. Organizations such as the severe adverse event consortium (SAEC) and Duke University are collaborating on identifying genetic risk factors for CIA via genetic association studies (http://www.genomeweb.com/dxpgx/saec-duke-collaborate-rare-variants-adverse-events-research). However, due to the rarity of suitable patients, such an approach requires global collaboration. Even if some statistically significant SNPs are identified by using genome wide association studies [5], [6], identifying the causal mechanism of such SNPs and using them in prediction models still presents a challenge. Instead of the traditional association study, we proposed an alternative computational methodology to identify the genetic risk factors for CIA, by identifying the known risk genes, explaining the relevant mechanism by observing chemical-protein interactions and providing a “most likely” candidate list [7] for pharmacogenetic and pharmacogenomic studies [8].

Drug-induced agranulocytosis is a form of idiosyncratic drug reaction (IDR). It is dose independent and is a form of serious adverse drug reaction [9], [10], [11]. One of the major causes of IDR is unexpected drug-protein interactions in human proteins [12], [13], [14], [15], [16], [17], [18]. Olanzapine (OLZ) is a CLZ analog, but has inferior efficacy in treating schizophrenia. It is reported to cause much less agranulocytosis compared with CLZ [19], [20], [21], a fact that is also confirmed in our statistical test (Fisher's exact test p = 8.2E-21, Table 1). Differences in their interaction profile towards human proteins (off-targets) might explain the etiology of CIA. Hence we hypothesized that if a human protein tends to be targeted by CLZ but not OLZ, the protein should be regarded as the candidate mediator of CIA, and the genes sharing a biological function with the off-targets (off-system, short for ‘off-target associated system’) should also display expression perturbation in cell lines treated by the drug. For example, we identified from a 410 protein target set retrospectively that Hsp70 protein as the off-target of CLZ but not OLZ, and that genes sharing the biological function with *HSPA1A* (Hsp70's gene) or acting as neighbors in Human Protein Reference Database (HPRD), a protein-protein interaction (PPI) database, with *HSPA1A* were found up-regulated in cell lines treated by CLZ. Another hypothesis is that if a protein target is preferably targeted by all drugs causing agranulocytosis (case) but not targeted by the agranulocytosis- drugs (control), the protein is a candidate mediator of the agranulocytosis. Using this hypothesis, we identified *NQO2* gene as the candidate gene of agranulocytosis.

**Table 1:** Test for the difference of the agranulocytosis report rate between clozapine and olanzapine in the FDA adverse event reporting system (AERS).

	Clozapine	Olanzapine
Agranulocytosis Reports	185	16
Total Reports	16813	11304
Ratio of Agranulocytosis Report (%)	1.1	0.14
p**_CLZ-OLZ_** *		8.2E-21

*Chi-square test for the equal rate of agranulocytosis between CLZ and OLZ. AERS records were updated in September, 2009.

## Results

### Preparing proteins and chemicals for chemical-protein interactome

**Figure 1:**
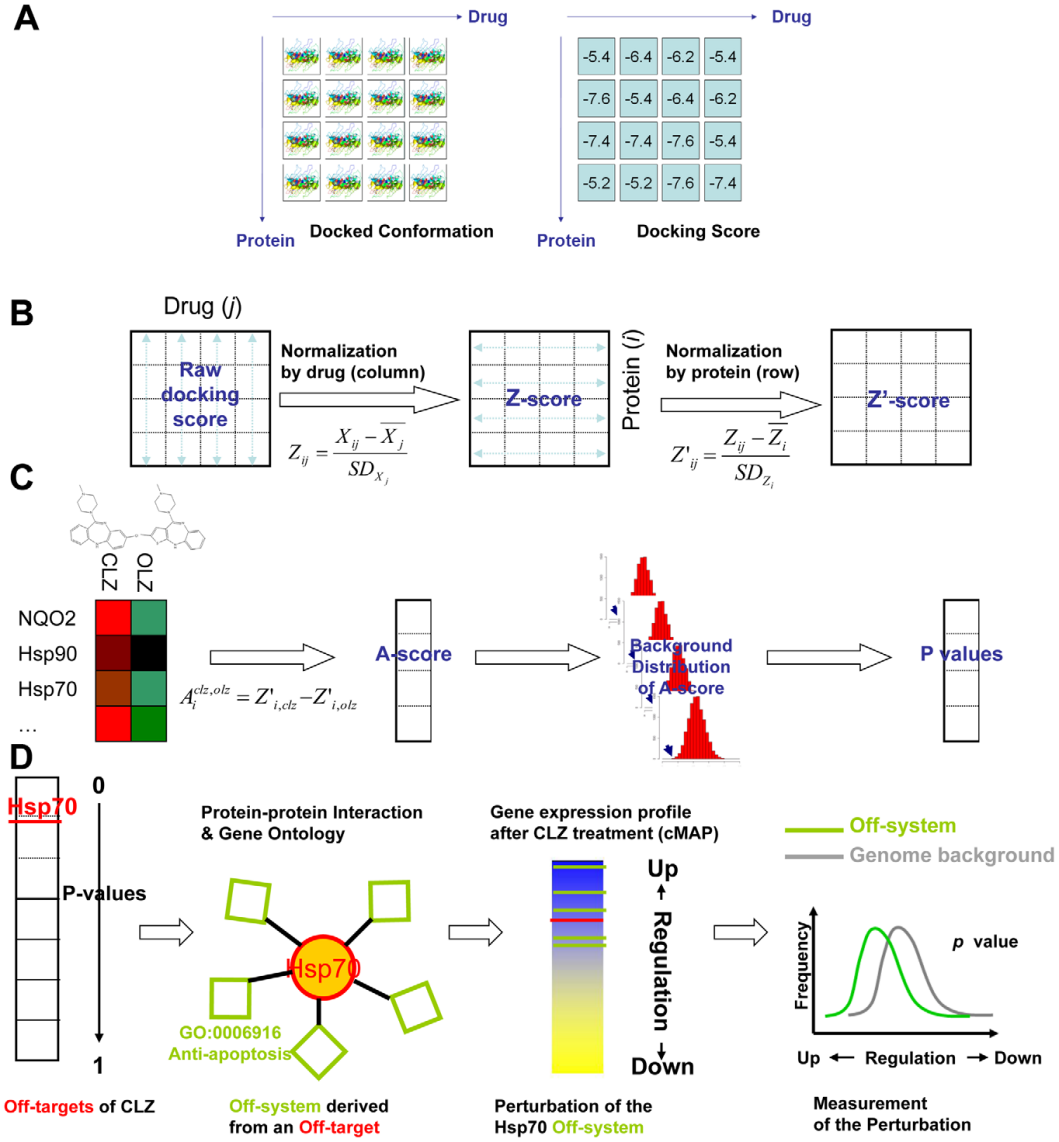
Workflow of construction and mining of the binomial antithesis chemical-protein interactome (CPI). (a) Binding conformations and raw docking scores were derived from the CPI with each column representing the drug molecule and each row representing the protein. (b) The 2DIZ transformation was applied to the CPI comprising 255 drugs and 410 protein pockets. (c) The OLZ and CLZ columns were extracted from the CPI where their Z′ score differences for each protein were measured by A-scores. The *p* values for each achieved A-score were calculated by simulating a random background. (d) Proteins were ranked according to their *p* values. In this case, Hsp70 was selected, proteins belonging to the same biological function (anti-apoptosis system or Hsp70's neighbor in HPRD network) were selected and then their expression changes in CLZ treatment were investigated (green bars indicated the rankings of the Hsp70 related genes when ordered by the change after CLZ treatment) and tested for significance by randomly selecting the same probe number in the genome background for permutation.

To identify unexpected drug-protein interactions, we utilized chemical-protein interactome (CPI) [13], [22], [23], which gives a score array generated by docking a panel of drug molecules across a set of human proteins. A CPI delivers two types of information, the binding conformation and the binding strength (Fig. 1a). It can be constructed via wet lab techniques [24], [25], [26], [27], but the most convenient way is to generate an *in silico* CPI. We used the DOCK [28] program to evaluate the chemical-protein interaction strength because it is an open-source software and had been widely used along with its success in identifying the unexpected chemical-protein interactions.

To prepare an unbiased protein set, we utilized a pocket set comprising 410 human protein pockets (381 unique proteins, Table S1), representing all the available human protein structure models from third-party target structural databases. The ligand binding pockets on each protein were then processed manually for docking preparation (see **Methods**).

We then mined from literature and the FDA adverse event reporting system (AERS) the drugs that were reported to cause agranulocytosis (case) or not cause agranulocytosis (control, Fig. S1a), aiming at identifying proteins tend to be targeted by case but not control drugs (red dashed rectangle in Fig. S1b). According to our criteria (**Methods**), there were 39 case and 15 control drug molecules selected for agranulocytosis, including the parent drug and their major metabolites and isomers. The control drugs did not share significant 2D structure similarity (Fig. S2), their indications covering a broad therapeutic categories (covering nine 1^st^ level of ATC codes). To generate a comprehensive distribution of docking scores for each protein across many drug molecules, we also incorporated other drug molecules. Although for effective performance and classification, a larger data set should be used [22], e.g., all the FDA approved drugs), we restricted our analysis to drug molecules from our former studies because of the CPU time for array docking. Thus, a total of 255 drug molecules, including the CLZ and OLZ, were selected for docking (Table S2).

### Constructing the chemical-protein interactome

Here 255 chemicals were docked into the 410 human proteins using DOCK, generating a docking score matrix of 255×410 elements. A 2-directional Z-transformation (2DIZ) [23] was then applied to transform the raw docking score into a Z′-score, extending the multiple active site corrections concept [29]. The docking scores were normalized by each drug and then by each protein (Fig. 1b), thus the “endogenous” variance among proteins, such as the free energy variation across the binding pockets, has been normalized and contribute almost zero to the variance of the Z′-scores (Table S3). The major contributions of the variance are from the chemical effects and the chemical-protein interactive effects after the 2DIZ, which means that each chemical can ‘fish’ its targets only based on Z′-score without noises from the “endogenous” variance among proteins.

### Binomial antithesis CPI between CLZ and OLZ

A basic assumption in using antithesis binding profile from CPI between CLZ and OLZ is that, 1) the two drugs are broadly similar in their effects, except for some side-effects, such as agranulocytosis, and that therefore, apart from some minor differences, their overall protein binding profile should be similar; 2) these minor differences in protein binding profile are highly likely to be associated with CIA. To verify the comparability between CLZ and OLZ, we calculated the Pearson's correlation coefficients (PCC) between Z′-score vectors of CLZ and OLZ across all 410 human proteins (with missing values removed). All four CLZ-OLZ pairs (2 CLZ ionization states×2 OLZ ionization states) obtained high positive PCC values (Fig. S3a). Their mean PCC value was distinctly higher (p = 0.0009 for permutation test in Fig. S3b). The high correlated protein binding profiles of CLZ and OLZ underlined their structural and pharmacological similarity, which also indicated the structural variability of all 255 drug molecules in the construction of the CPI. We therefore hypothesized that the proteins exhibiting different binding affinity against CLZ and OLZ might account for the agranulocytosis risk of these two analogs.

In order to identify the minor distinctions, we defined the antithesis score (A-score) for protein i as the Z′-score difference between CLZ and OLZ towards protein i,
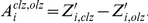



We also calculated the probability of an A-score less than 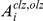 between two randomly selected drug molecules among 255 molecules at protein i (Fig. 1c), which could be expressed as,
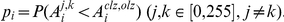



We performed permutations for each target by randomly selecting drug-pairs and calculating their A-scores 10,000 times. Here the p value was the one-tailed probability when the A-score of the drug-pair was less than that of the CLZ-OLZ pair. Targets with p value less than the 0.05 cutoff are shown in Table 2. For the four CLZ-OLZ pairs, we chose only the pair that recalled most known CIA related genes reported in the genetic association studies.

**Table 2:** Candidate off-targets/-systems prioritized from binomial antithesis CPI between CLZ and OLZ.

PDB ID#	Target Name	Gene Name	Z′ (CLZ)*	Z′ (OLZ)	A-score	p value for CPI	Role	Sys. Regulation	p value for Sys. perturbation
1CBS	Cellular retinoic acid-binding protein 2	CRABP2	−0.922	1.653	−2.575	0.000			
1D1T	Alcohol dehydrogenase class 4 mu/sigma chain	ADH7	−1.191	1.525	−2.716	0.000	OR		
1IHI_1	Aldo-keto reductase family 1 member C2	AKR1C2	−0.781	2.545	−3.326	0.000	OR		
1IHI_2	Aldo-keto reductase family 1 member C2	AKR1C2	−1.605	1.023	−2.628	0.000	OR		
1OIZ	Alpha-tocopherol transfer protein	TTPA	−1.269	1.171	−2.440	0.000			
2E8A	Heat shock 70 kDa protein 1	HSPA1A/HSPA1B	−1.381	0.150	−1.531	0.001		up	0.0289
1D2V	Myeloperoxidase	MPO	−2.753	−0.646	−2.107	0.005	OR		
1DB1	Vitamin D3 receptor	VDR	−0.660	0.748	−1.409	0.012		up	0.0139
1MRQ_2	Aldo-keto reductase family 1 member C1	AKR1C1	−2.034	0.123	−2.158	0.016	OR		
1MRQ_1	Aldo-keto reductase family 1 member C1	AKR1C1	−1.036	0.601	−1.637	0.021	OR		
1DHT	Estradiol 17-beta-dehydrogenase 1	HSD17B1	−1.822	0.158	−1.980	0.021	OR		
1MUO	Serine/threonine-protein kinase 6	AURKA	−1.136	0.529	−1.665	0.027		up	0.0749
1VJ5	Epoxide hydrolase 2	EPHX2	−1.088	0.228	−1.315	0.027			
4GTU	Glutathione S-transferase Mu 4	GSTM4	−0.749	1.060	−1.809	0.036	GT	down	0.2758
1HDR	Dihydropteridine reductase	QDPR	−1.469	0.561	−2.030	0.038	OR		
1YB5	Quinone oxidoreductase	CRYZ	−1.212	0.284	−1.496	0.039	OR		
1CM8	Mitogen-activated protein kinase 12	MAPK12	−1.202	0.301	−1.503	0.039		up	0.1238
1XF0_2	Aldo-keto reductase family 1 member C3	AKR1C3	−0.865	0.441	−1.306	0.041	OR	up	0.0113
1HMR	Fatty acid-binding protein, heart	FABP3	−0.826	0.270	−1.095	0.046		down	0.1968

# An entry name that ends with a number represents the pocket number of its PDB structure.

*The smaller Z′-score represents a higher theoretical interaction strength.

In the “Role” column, OR and GT indicate oxidoreductases and gluthathione metabolism related proteins, respectively.

### Multiple antitheses CPI between case and control drugs

A chemical-protein interaction with a Z′-score less or greater than −0.48 was defined as interactive or not interactive, respectively. As indicated in our previous training set [22], Z′-scores above such cutoff captured 70% of the true bindings and were enriched more than three-fold as compared with the false binding. For protein i, *a*_i_, *b*_i_, *c*_i_, and *d*_i_, denoting the number of interactive (*a*_i_ or *b*_i_) and not interactive (*c*_i_ or *d*_i_) by case or control drug molecules, respectively, were counted and the relative ratio (*RR*) was calculated as follows,
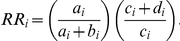



To identify proteins preferentially interacting with the case drugs, we performed Fisher's exact tests for each protein. The significance (one-sided) for each of the protein pockets with *RR* value exceeding one were computed and were used as a measure to prioritize the potential protein mediating agranulocytosis. Table 3 shows protein targets with *p* values less than 0.05.

**Table 3:** Targets selected from multiple antitheses CPI between case and control drugs with *p* value less than 0.05.

PDB ID^a^	Target Name	Gene Name	a	b	c	d	RR	p value	Role
1I10	L-lactate dehydrogenase A chain	LDHA	21	1	18	14	1.697	0.002	OR
2HGS_2	Glutathione synthetase	GSS	24	2	15	13	1.723	0.002	GT
2HRB	Carbonyl reductase NADPH 3	CBR3	17	0	22	15	1.682	0.002	OR
1KBQ	NAD(P)H dehydrogenase quinone 1	NQO1	16	0	23	15	1.652	0.002	OR
1EEM	Glutathione S-transferase omega-1	GSTO1	19	1	20	14	1.615	0.004	GT
1SG0_2	Ribosyldihydronicotinamide dehydrogenase quinone	NQO2	14	0	22	15	1.682	0.005	OR
1G0X	Leukocyte immunoglobulin-like receptor subfamily B member 1	LILRB1	15	0	24	15	1.625	0.005	
2AHE	Chloride intracellular channel protein 4	CLIC4	14	0	24	15	1.625	0.005	
1DIA	Formyltetrahydrofolate synthetase	MTHFD1	14	0	25	15	1.600	0.006	OR
11GS	Glutathione S-transferase P	GSTP1	18	1	21	14	1.579	0.009	GT
1FIE	Coagulation factor XIII A chain	F13A1	12	0	23	15	1.652	0.010	
1Q4O	Serine/threonine-protein kinase PLK1	PLK1	13	0	25	15	1.600	0.011	
1LJR	Glutathione S-transferase theta-2	GSTT2B	8	0	12	14	2.167	0.011	GT
1FPR	Tyrosine-protein phosphatase non-receptor type 6	PTPN6	13	0	26	15	1.577	0.011	
1HSO	Alcohol dehydrogenase 1A	ADH1A	13	0	26	15	1.577	0.011	OR
1IHI_1	Aldo-keto reductase family 1 member C2	AKR1C2	20	2	19	13	1.531	0.014	OR
1SG0_1	Ribosyldihydronicotinamide dehydrogenase quinone	NQO2	16	1	22	14	1.540	0.020	OR
1IHI_2	Aldo-keto reductase family 1 member C2	AKR1C2	16	1	23	14	1.514	0.021	OR
1D5R	Phosphatidylinositol-3,4,5-trisphosphate 3-phosphatase and dual-specificity protein phosphatase PTEN	PTEN	11	0	26	15	1.577	0.022	
1XWK	Glutathione S-transferase Mu 1	GSTM1	11	0	26	15	1.577	0.022	GT
1TDI	Glutathione S-transferase A3	GSTA3	11	0	27	15	1.556	0.023	GT
5GAL	Galectin-7	LGALS7 | LGALS7B	11	0	27	15	1.556	0.023	
1W7N	Kynurenine–oxoglutarate transaminase 1	CCBL1	11	0	28	15	1.536	0.024	
2AB6	Glutathione S-transferase Mu 2	GSTM2	11	0	28	15	1.536	0.024	GT
1MQ0	Cytidine deaminase	CDA	15	1	24	14	1.484	0.024	
1OAT	Ornithine aminotransferase, mitochondrial	OAT	15	1	24	14	1.484	0.024	
1ANG	Angiogenin	ANG	12	0	27	15	1.556	0.024	
1J8F	NAD-dependent deacetylase sirtuin-2	SIRT2	12	0	27	15	1.556	0.024	
2J0D	Cytochrome P450 3A4	CYP3A4	12	0	27	15	1.556	0.024	OR
1XF0_1	Aldo-keto reductase family 1 member C3	AKR1C3	19	2	19	13	1.524	0.027	OR
1I0Z	L-lactate dehydrogenase B chain	LDHB	19	2	20	13	1.493	0.028	OR
2BX8_3	Serum albumin	ALB	17	2	19	13	1.507	0.028	
1GOS_2	Amine oxidase flavin-containing B	MAOB	20	3	16	12	1.522	0.030	OR
1UKI	Mitogen-activated protein kinase 8	MAPK8	18	2	21	13	1.457	0.031	
2BXF	Serum albumin	ALB	18	2	21	13	1.457	0.031	
5P21	GTPase HRas	HRAS	14	1	24	14	1.478	0.041	
1PIN	Peptidyl-prolyl cis-trans isomerase NIMA-interacting 1	PIN1	9	0	24	15	1.625	0.041	
1H0C	Serine–pyruvate aminotransferase	AGXT	14	1	25	14	1.456	0.043	
1A5Y	Tyrosine-protein phosphatase non-receptor type 1	PTPN1	14	1	24	13	1.439	0.043	
1QMV	Peroxiredoxin-2	PRDX2	9	0	27	15	1.556	0.044	OR
1BJ4	Serine hydroxymethyltransferase, cytosolic	SHMT1	10	0	29	15	1.517	0.046	
2CYK	Interleukin-4	IL4	10	0	29	15	1.517	0.046	
3DYD	Tyrosine aminotransferase	TAT	10	0	29	15	1.517	0.046	
1HE5_2	Flavin reductase	BLVRB	9	0	30	15	1.500	0.049	OR

a An entry name that ends with a number represents the pocket number of its PDB structure.

In the last column, OR and GT indicate oxidoreductases and gluthathione metabolism related proteins, respectively.

### Retrospective study of the genetic risk factors of CIA

Besides human leucocytes antigen (HLA) markers, three CIA susceptible genes have been identified in genetic association studies [30], namely *HSPA1A* [31], *TNF* [32] and *NQO2* [33]. None of the HLA proteins were included in our pocket set since they did not meet our criteria of choosing protein pockets. Proteins coded by these three susceptibility genes all happen to be included in our pocket set comprising third party targetable protein databases (Table S1).

**Figure 2:**
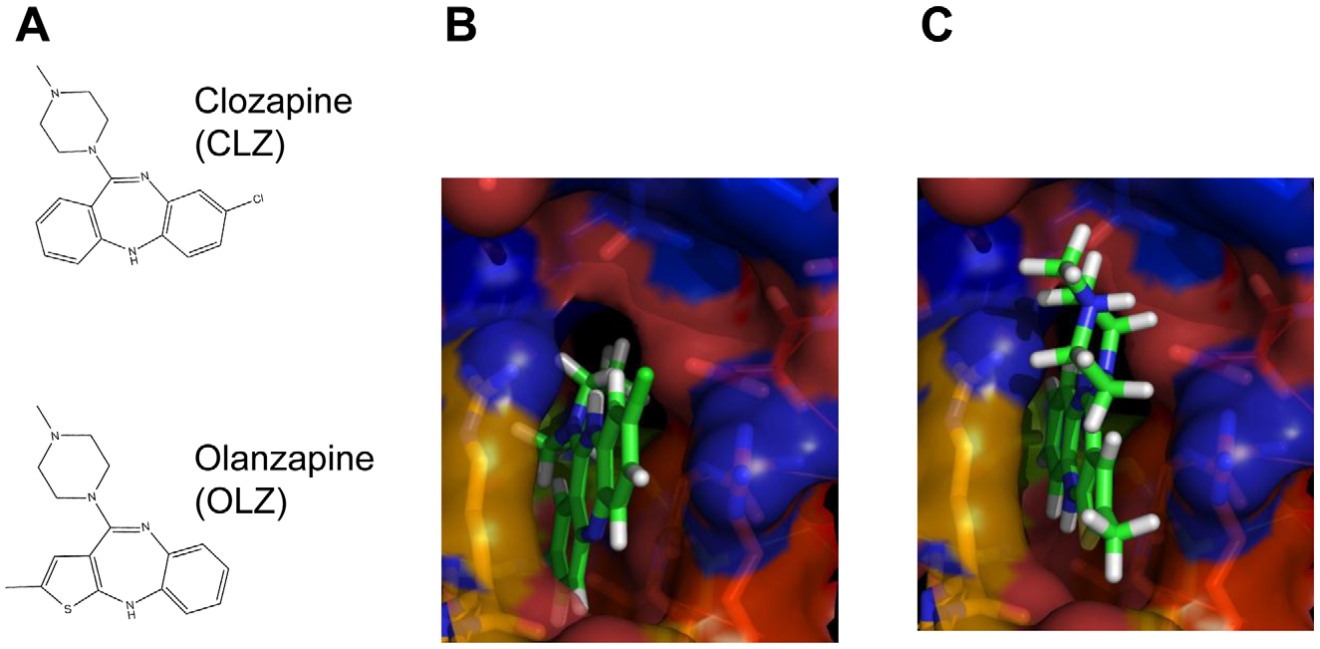
Structural comparison of clozapine and olanzapine towards HSP70 protein. (a) The structural difference between CLA and OLZ. (b, c) Binding conformation of CLZ and OLZ towards the Hsp70 ligand binding pocket. The whole molecule of CLZ binds deep into the pocket, leaving the chlorine atom at the surface. However, the major part of the OLZ molecule is not accommodated in the deep pocket due to the steric hindrance of the methyl on the heterocycle of OLZ. The figures were drawn using PyMOL.

*HSPA1A* codes the heat shock 70 kD protein 1 (Hsp70 protein, PDB ID: 2E8A) and has been reported in a high profile journal to be associated with CIA with its causality in CIA discussed [31]. It is also well known for its druggability in antitumor drugs [34], which in general, cause the death of the cell. The gene was prioritized in our binomial antithesis CPI (Table 2). Significant binding strength differences between CLZ and OLZ towards Hsp70 were identified with the binding conformations visualized in Fig. 2. The CLZ molecule fits deeply into the Hsp70 pocket (Fig. 2b). By contrast, the methyl group of OLZ was difficult to accommodate in the narrow pocket using the similar binding pose as CLZ (Fig. 2c).

**Figure 3:**
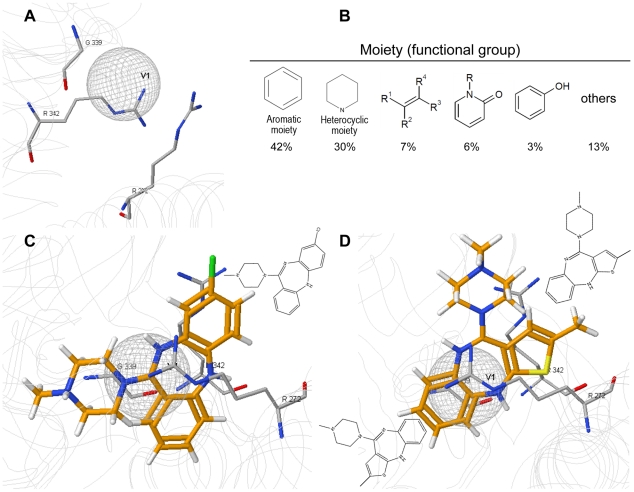
Site-moiety map analysis of the Hsp70 pocket. (a) The van der Waals-interacting anchor site with three essential residues (R272, R342 and G339). (b) Percentages of the functional group among all docked drug molecules. The binding conformation of CLZ (c) and OLZ (d) towards this site. The molecule directions are also indicated in the 2D molecule structures at the top right corner of (c, d). Bottom left of (d) shows the direction of the OLZ as if it wants to interact using the same pattern as CLZ but significant steric hindrance makes insertion into the pocket in this way difficult.

We further performed the site-moiety map analysis [35] of the Hsp70 pocket by examining the moiety preferences of the docked ligands and the physicochemical properties of the pocket. One van der Waals-interacting anchor site was identified with three essential residues (R272, R342 and G339, Fig. 3a). Among the docked drug molecules, most used the aromatic moiety or conjugated bonds to interact with this center (Fig. 3b). Theoretically, both CLZ (Fig. 3c) and OLZ (Fig. 3d) should have been capable of insertion into this pocket, however, the methyl on the OLZ molecule made it difficult to hold the same binding direction as that of the CLZ (see molecule structures in Fig. 3c, d). The CLZ molecule was inserted deep into the pocket and used most of its conjugated ring system to interact with the R272 and R342 via π-π interaction. Compared with CLZ, OLZ could not use the majority of its conjugated system due to steric hindrance caused by his methyl group. The above findings add evidence to the hypothesis that the Hsp70 protein was the off-target of CLZ but not of OLZ.

Ribosyldihydronicotinamide quinone dehydrogenase (coded by *NQO2*; PDB ID: 1SG0), the known risk gene for CIA, was prioritized from the multiple antitheses CPI (Table 3), together with other 44 proteins with p value less than 0.05. The protein was preferably targeted by the case but not the control drugs. The Kolmogorov-Smirnov test of the Z′-scores between cases and controls showed significant differences on two pockets (p = 0.002 and p = 0.004 for pocket 1 and 2, respectively). As for the binomial antithesis CPI, NQO2 protein ranked 37^th^ among the 410 proteins (top 9%) when ordered by *p* value. Although the *p* value did not exceed the 0.05 threshold, the A-score was −1.18, indicating that there were still differences between the interaction strength of CLZ and OLZ towards this protein.

Myeloperoxidase and NADPH-oxidase are functionally involved in the pathogenesis of the drug-induced agranulocytosis [36], [37]. Myeloperoxidase (PDB ID: 1D2V) was found in Table 2 whereas two oxidoreductases using NADPH as the co-enzyme, namely Carbonyl reductase NADPH 3 (2HRB) and NAD(P)H dehydrogenase quinone 1 (1KBQ) were found in Table 3.

We also investigated the genetic polymorphisms of genes coding Hsp70, NQO2 protein, Myeloperoxidase and NADPH-oxidase. Some nonsynonymous single nucleotide polymorphisms (SNPs) were identified but none of these was found to affect the ligand binding pockets.

### Clozapine perturbation on the Hsp70-associated system

Besides bindings between chemicals and proteins, the drug-target relationship may also be reflected in the expression changes of genes related to the off-target associated system [38] after chemical treatment. If the mRNA expression of a set of genes related to off-target *X* is significantly changed after drug treatment, both target *X* and the associated system *X* could corroborate each other for their roles in the adverse reaction. Since Hsp70 was identified as the putative off-target of CLZ, we sought to investigate whether the CLZ treatment resulted in perturbation of Hsp70 and the related gene system. We analyzed the data from Connectivity Map (cMAP) [39], a collection of gene expression data from drug-treated human cell lines on Affymetrix U133A microarrays. Cells were treated by particular drug and vehicle respectively to measure the change of gene expression. One such drug-vehicle pair was defined as an instance. For all 6,100 instances, 22,283 probes were ranked by fold-change values with higher fold-change ranked at the top (close to rank 1), forming a 22283×6100 matrix. We recruited all four instances (instance 1170, 1289, 2689 and 6188) performed on the human promyelocytic leukemia (HL60) cell line to specifically address the drug effect of CLZ on the leukocytes. Instances performed on other cell lines were also investigated.

**Figure 4:**
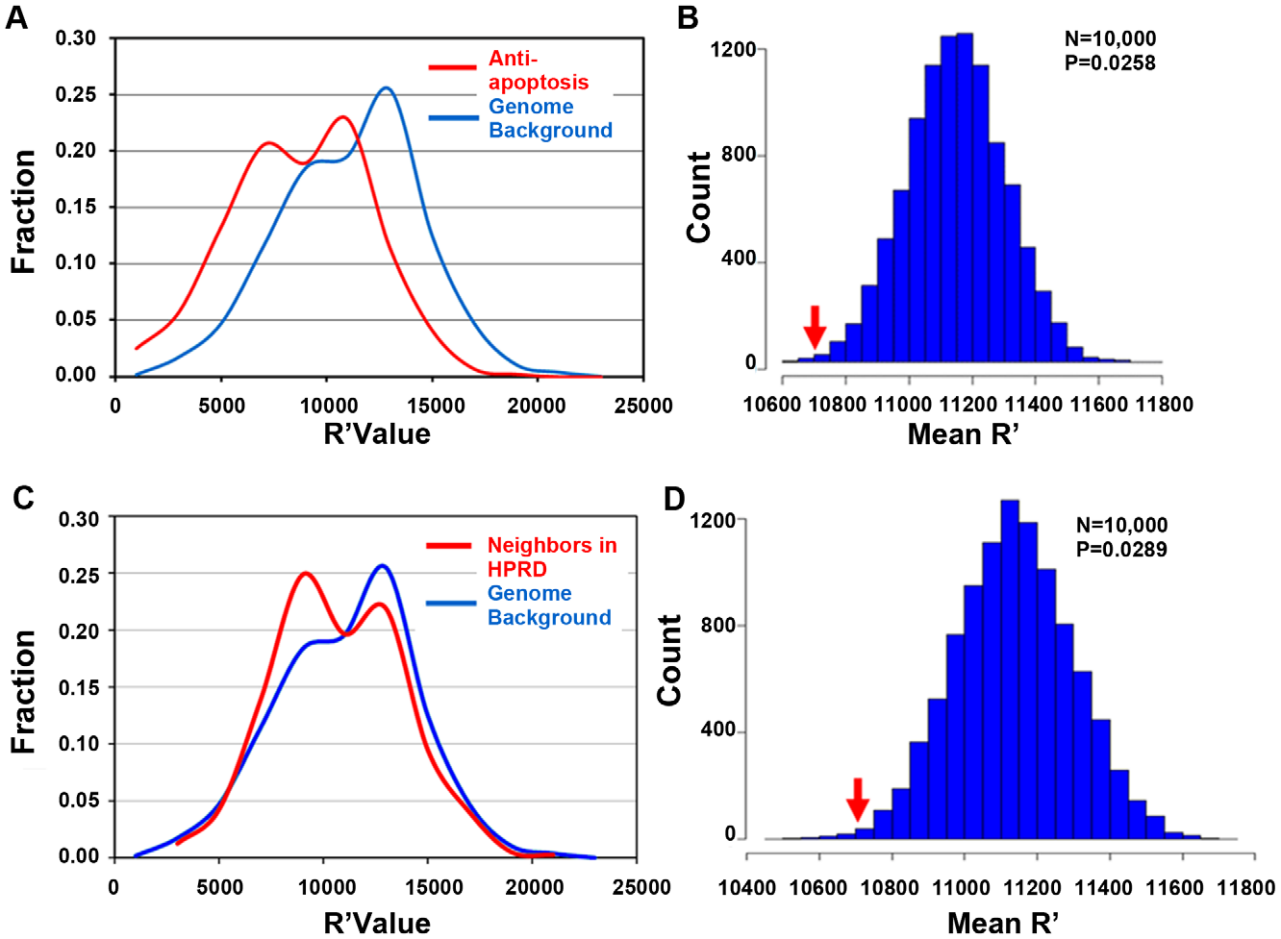
Clozapine disturbance effect towards the Hsp70 systems. Compared with the genome background, genes related to anti-apoptosis (a) or Hsp70's neighbor in HPRD network (c) were generally up regulated in CLZ treated HL60 cell lines, in terms of higher R′ value. The mean R′ of anti-apoptosis (b) or Hsp70's neighbor in HPRD network (d) related gene system was significantly higher than randomly selected genes in the genome background simulated by permutation test.

We then manually extracted genes related to *HSPA1A* in Gene Ontology (GO) (Fig. 1d) [40]. *HSPA1A* was associated with 7 GO terms in the biological process. As agranulocytosis is basically the death of neutrophil and is known to be correlated to apoptosis pathways [41], we choose the term “anti-apoptosis” (GO:0006916) to characterize the role of *HSPA1A* in CIA. We selected all human genes linked to this term that collectively represented the Hsp70 off-system. These genes were mirrored to probes on microarray (439 probes corresponding to 235 genes). For each probe, we calculated the average rank of the probe across four CLZ instances (R′ rank), with higher R′ (closer to rank 1) indicating generally up regulated status and lower R′ down regulated status. We compared the R′ of the Hsp70 system and other genes on the U133A probe set. The anti-apoptosis system exhibits an R′ distribution quite distinct from that of the genome background (Fig. 4a), with significantly higher mean R′ than the random 235 gene set (258 out of 10000 sets showed higher R′, p = 0.0258 for permutation test, Fig. 4b). The general up regulation of Hsp70 related genes indicates that CLZ treatment clearly changes the bioactivity of the Hsp70 system in human HL60 promyelocytic leukemia cells. The Hsp70 off-system's perturbation was further confirmed using *HSP1A1*'s ‘neighbor’ in HPRD [42] network) following the same procedure as for investigating the anti-apoptosis system (Fig. 4c,d). Both GO term-based off-system and the PPI-based off-system corroborate the important role of Hsp70 in CIA. The cMAP also contains breast cancer cell line MCF7 and human prostate cancer cell line PC3, however, none of the perturbation of the Hsp70 system could be detected in these two cell lines. The significant perturbation could not be detected on other six GO terms of *HSP1A1*.

### Two-dimensional elucidation of the off-targets and the off-systems after clozapine treatment

The drug-(off) targets interaction and the gene expression change are the molecular events at two different dimensions after drug treatment. To get an overview of the systems perturbation of the off-targets prioritized in Table 2, we investigated the PPI-based off-systems for them. We did not choose the GO term-based off-systems because each gene was related to multiple GO terms, and it was difficult to objectively choose the appropriate GO terms related to agranulocytosis. Furthermore, using PPI-based off-systems to study the drug's perturbation on the biosystems has been proved to be applicable [43]. Among 17 off-systems, three were found to be significant perturbed with a permutation p value less than 0.05 (Table 2), including Hsp70 off-system.

**Figure 5:**
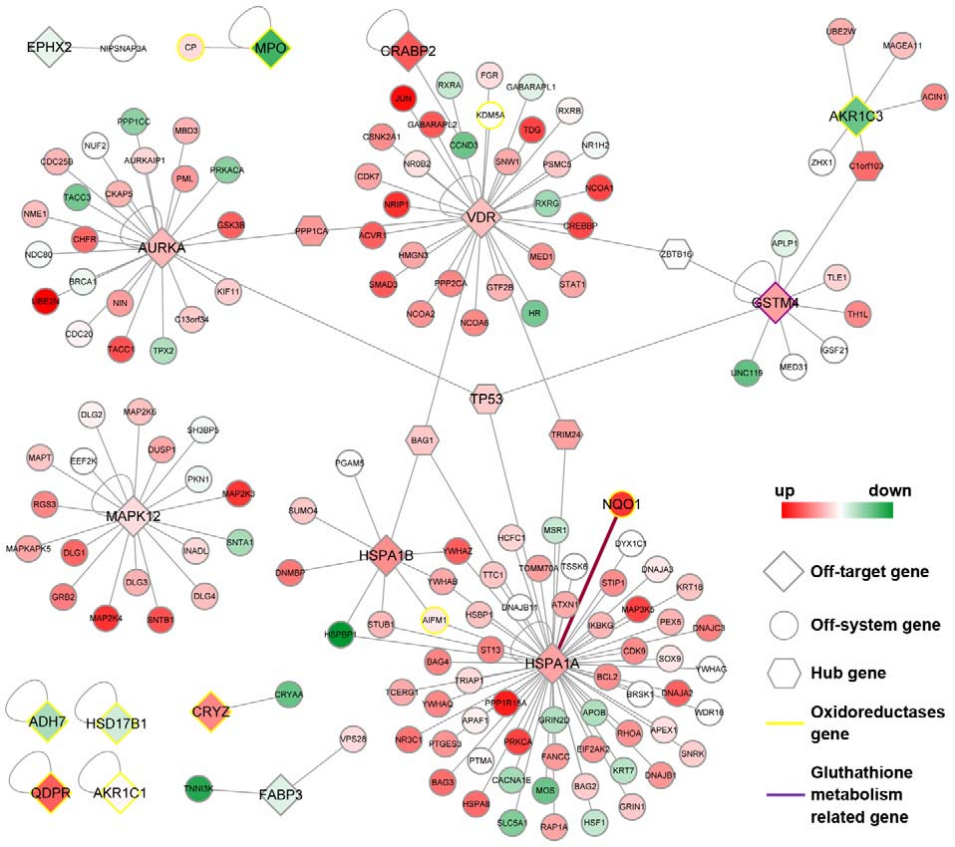
Off-targets and their off-systems' perturbation after clozapine treatment. The off-targets, the genes involved in the PPI-based off-systems and the hub genes are in diamond, circle and hexagon shape, respectively. The PPI information from HPRD contains binary PPI and protein complex, and only the former information is visualized in this figure for brief. Red/green indicates the up-/down-regulation of the gene expression after clozapine treatment. Oxidoreductases and gluthathione metabolism related protein are in yellow and purple edges, respectively. The interaction between *HSPA1A* and *NQO1* was highlighted in red line.

The PPI-based off-systems were then visualized in Fig. 5, where the gene expression perturbation ‘landscape’ of the off-systems was shown. These off-systems were found to be connected by several hub nodes, such as apoptosis associated gene (*TP53*), the gene coding Bcl-2-binding protein (*BAG1*) and the transcriptional regulator of vitamin D3 receptor (*TRIM24*) *et al.* Interestingly, *NQO2* was also found to be involved in *HSPA1A* off-system and significantly up-regulated after CLZ treatment. Besides preferably inhibited by CLZ, most of the oxidoreductases were found down-regulated or remain unchanged after CLZ treatment. The whole picture demonstrated that the impact of CLZ on the HL60 cell line is reflected on the up-regulation of the anti-apoptosis systems and the inhibition or the down-regulation of the oxidoreductases.

### Perspective investigation of the predicted genetic risk factors of CIA

Interestingly, oxidoreductases were found to be significantly enriched in prioritized proteins. For example, quinone oxidoreductase (PDB ID: 1YB5), an isozyme of the NQO2 protein, also appears in Table 2. Seventy out of 410 protein pockets (17%) were oxidoreductases (Table S1). However, as Table 2 shows, oxidoreductases were significantly enriched (10 out of 19, 53%, Fisher's exact test p = 6.6E-4). Among targets prioritized by multiple antitheses CPI (Table 3), 15 out of 44 pockets (34%) belonged to oxidoreductases (p = 7.9E-3). In addition, only 12 out of 410 protein pockets (3%) were related to glutathione metabolite, which plays key role in antioxidation. However, as Table 3 shows, 7 out of 45 (16%) were significantly enriched (p = 1.2E-3).

## Discussion

Identification of off-targets has potential application in drug repurposing [44], [45] and personalized medicine [13], [46]. Compared with the similarity ensemble approach [47] and the naive Bayesian classifiers approach [48] to off-target identification, both of which build new drug-protein connections within the space of the known therapeutic target, the chemical-protein interactome approach is a step towards analyzing the entire human proteome, although the available human protein structrome is limited. Several of the pocket comparison algorithms have also tried to explore the off-target spaces facing the entire human proteome [15], [17], or tried to map the off-targets onto the pathways [49] or the metabolic network [50], but our study is the first one examining the system's perturbation in terms of both the off-target identification and the off-system's gene expression change, providing candidates for pharmacogenetic and pharmacogenomic studies, respectively. Further work may combine the off-target and the off-system in elucidating and predicting adverse drug reactions.

In the retrospective studies, the antitheses CPI recalled the accredited susceptible genes for CIA. As a complement to genetic association studies [6], the CPI reveals the possible mechanism of the CIA based on the drug-protein interaction, the primary step in drug reaction. The difference between the interaction conformation and the interaction strength of CLZ and OLZ towards the off-targets could account for the difference in patients' susceptibility to agranulocytosis. Since none of the nonsynonymous SNPs was found around the ligand binding pocket of the four proteins reported to be involved in CIA, we deduced that individual differences in CIA susceptibility could be explained by a variation in the expression level of the protein. In fact, *NQO2* was found to have lower expression levels in CIA susceptible patients [33]. The lower expression level in this detoxification enzyme could make the patient more sensitive to the drug. It is also reasonable to expect subsequent discoveries (e.g. some genotypes correlated to *Hsp70* or *NQO2* expression level) supporting the CLZ off-target hypothesis, which could lead to biomarker development at genotype and gene expression level [51] in CLZ therapy.

The reactive oxygen hypothesis is one of the major hypotheses of agranulocytosis etiology [37]. In our results, CLZ and other drugs causing agranulocytosis tended to affect the oxidoreductases, which play an important role in reactive oxygen clearance. For example, NQO2 protein and myeloperoxidase are key enzymes in the detoxification of active radicals thus protecting the cells from drug-induced oxidative and electrophilic stress [52]. Furthermore, alpha-tocopherol transfer protein is a prioritized target of clozapine (Table 2). Blocking the transferring of tocopherol, which is a strong endogenous antioxidant [53], may also explain clozapine's impact on the detoxification system. Clozapine can be oxidized to reactive nitrenium ions [54], which preferably reacts with sulfhydryl and is detoxified by glutathione. In our results, glutathione related enzymes were significantly enriched in the CPI, implying that the drug causing agranulocytosis not only affected the detoxification system of oxidoreductases, but might also interfered in the glutathione system, which is essential to the detoxification of the major metabolites of CLZ.

Besides the unexpected drug-protein interactions, the expression change of the off-system may explain CIA etiology. The perturbation of anti-apoptosis genes by CLZ treatment reflects the fact that CLZ disturbs cell death pathways by binding with Hsp70, and the general up regulation of anti-apoptosis genes can be explained as a feedback towards elevated apoptotic stress mediated by Hsp70 and the anti-oxidation system, since the inhibition of oxidoreductases and the perturbation of oxidoreductase system is a well known mediator of apoptosis [55]. By breaking the balance of oxidation and reduction, CLZ can stimulate apoptosis via Hsp70 inhibition and enhanced oxidative stress. Along with the CPI results, biological effects of CLZ further support the hypothesis that Hsp70 and oxidoreductases together with their respective system serve as the off-targets(-systems) of CLZ and potentially mediate CIA. Since HL60 is derived from peripheral blood leukocytes, which is a representative cell model for the immune system, the finding of the systems perturbation in HL60 cells but not in MCF7 (breast cancer) and PC3 (prostate cancer) cell lines strengthens the antiapoptosis and the oxidoreductases systems' function in immune related events. In summary, 53% and 34% of prioritized proteins from the CPI are oxidoreductases, and 16% of the proteins are related to gluthathione metabolism. These findings suggest a much higher participation of the detoxification/antioxidant systems in drug-induced agranulocytosis than previously thought and the off-targets/-systems identified in this study can represent candidates for biomarker development in wet-lab experiments and pharmacogenetic/pharmacogenomic screening in the future.

However, the 410 binding pocket set is a limited representation of the entire human proteome. For instance, it does not include any HLA proteins according to our target preparation criteria, which may be involved in agranulocytosis as a mediator of the immune etiology. Drug-HLA interaction was reported to be an important step determining the drug-HLA specificity in IDR [56]. In our previous study, we have built the abacavir-HLA-B*5701 interaction models for abacavir-induced hypersensitivity [13]. The identification of the drug-HLA interaction at the F-pocket of HLA molecules has been cited by several immunologists [57], [58]. Since HLAs have been identified as the key factors in IDRs [5], [6], [59], [60], the drug-HLA interactome will be systematically studied in future.

Identification of the related genes and the systems is the first step towards understanding and more importantly, predicting the IDR. The IDRs were regarded as unpredictable in response to compounds [61]. In this study, we argue that the IDRs are predictable, and the challenge of personalized medicine is not to predict adverse reaction for a compound but for a patient. The biomarkers could be either the genetic variations causing a binding affinity change of the drug towards the off-targets [62], [63], the expression level alteration of one gene [33], or the off-systems' perturbation. Our study demonstrates that beside polymorphisms around the binding pocket that alter the drug efficacy via a change in the binding affinity [64], [65], the off-system expression change could also determine individual variability towards the same drug, suggesting a new way of identifying biomarkers or constructing a prediction model for personalized medicine. Such an approach could also be applied to personalized drug repurposing [66], [67], [68], where the off-targets and the off-systems accounting for the new therapeutic area could also be patient specific.

Adverse drug reaction and the new indication are two ‘off-effects’ of the drug towards human being. So this study will also illuminate the drug repositioning by, 1) helping explain the mode-of-action of the serendipitous repositioned drugs via identifying their off-targets/-systems; 2) predicting the new use for existing drugs based on their interaction profiles with the off-targets and their perturbations on the off-systems. For example, one can recruit the case and the control molecular set for a particular indication. After identifying the off-targets/-systems using the methodology in this study, one can predict the indication of a new compound based on its impact on these newly identified off-targets/-systems.

## Methods

### Analysis of the adverse drug reaction report

The reports were downloaded from the FDA's AERS (http://www.fda.gov/Drugs/GuidanceComplianceRegulatoryInformation/Surveillance/AdverseDrugEffects/default.htm). This system tracks adverse events that are voluntarily reported but only the records from 2004 were freely available. All reports bearing CLZ and OLZ as the primary or secondary suspected drug were counted. The numbers of agranulocytosis cases were then counted for each drug. We performed Fisher's exact test to examine the frequency difference.

### Preparing the target set

Protein targets were obtained from third-party protein structure databases, including a drug adverse reaction target database [69], a drug-induced toxicity related protein database [70], a therapeutic target database [71] and a protein database for drug target identification [72]. Every pocket was examined manually when constructing the target set for DOCK according to the following criteria. First, the species should be confined to *Homo sapiens*; secondly, a co-crystallized ligand must be contained to indicate the targetable state of the protein; thirdly, the pocket should not contain missing residues. Spheres whose radii ranged from 1.1–1.4 Å were generated to fill in the pocket. A grid box was constructed 3–5 Å from the spheres. EC classifications of the enzymes were taken from the annotations of UniProt [73]. Finally, we achieved 410 protein pockets from 384 PDB entries, 74% of which have the resolution less than 2.5 Å.

### Choosing the cases and controls for multiple antitheses CPI

Drugs reported in the PubMed literature (up to September, 2009) as being associated with agranulocytosis were chosen as candidates and further examined in the AERS administered by the FDA/Center for Drug Evaluation and Research (http://www.fda.gov/Drugs/GuidanceComplianceRegulatoryInformation/Surveillance/AdverseDrugEffects/default.htm). All AERS raw data were downloaded from the FDA website and then placed in a relational database (MySQL 5.1). Accessible data were limited to the period from Jan 2004 to March 2009. In any adverse event report, only the primary or the secondary suspected drugs were regarded as linked to agranulocytosis. The candidates were only included if the number of reports exceeded 3. The candidates for control drugs were collected from AERS data, on condition that there were no reports of agranulocytosis. Candidates were then confirmed as control drugs only if they had never been co-cited with agranulocytosis in PubMed literature and the first 10 results of a Google search (up to September, 2009; with drug name AND “agranulocytosis” as query term). The major metabolites and the isomers of the drugs were also included. In the end, 39 case and 15 control drug molecules were selected for agranulocytosis endpoint. These 15 controls do not share significant 2D structure similarities. The SMILES code of the drugs and their derivatives was retrieved from PubChem. The 3D conformations of chemicals were simulated using CORINA. Charges and hydrogens of proteins and chemicals were added using Chimera [74].

### Choosing the background drug molecules

The background drugs were chosen from the molecules prepared in our previous studies, including anti-Alzheimer drugs [22], drugs referred to in the study by Lamb et al [39] on using the cMap, case and control drugs for rhabdomyolysis, cholestasis, deafness and Stevens-Johnson syndrome and QT prolongation [13]. A total of 255 drug molecules, including case and control drugs for agranulocytosis, were involved in constructing the CPI.

### Constructing the CPI

A CPI comprising 255 drugs towards 410 protein pockets was constructed using the DOCK [28] program controlled by Bash shell scripts. The parameters for docking corresponded to the default settings. The 2DIZ transformation [23] was performed where the docking score matrix was normalized first by one drug towards the 410 proteins then by one protein pocket towards the 255 drugs. The empirical threshold −0.48 of the Z′-score was set to distinguish binding and non-binding, based on the findings of the previous studies [22], [23].

### Permutation test for the PCC of CLZ-OLZ pairs

To determine the significance level of similarity between four CLZ-OLZ pairs (2 CLZ ionization states×2 OLZ ionization states) across their protein binding profile, we randomly recruited 10,000 sets with four drug pairs from all 255 drugs in the CPI, and identified 9 pairs with mean PCC not lower than the mean PCC of the four CLZ-OLZ pairs.

### Microarray data analysis

Suppose there are *n* genes sharing a specific GO term or linked to the same hub in the HPRD network. Each probe was independently ranked according to expression change for each instance in cMAP, with most up-regulated being at the top. For the cMAP instance # 1170, 1289, 2689 and 6188, which were the CLZ-treated instances, we calculated the mean rank R′ of each probe as
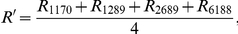
where R_1170_, R_1289_, R_2689_ and R_6188_ indicate the rank in instance 1170, 1289, 2689 and 6188, respectively. For evaluation on the perturbation status of a system, we randomly recruited 10,000 sets with *n* genes, obtaining *m* sets with mean rank higher than the object system. The p value was calculated as *m*/10000.

### Locating the polymorphism onto the proteins

Polymorphism information for the genes was retrieved from dbSNP [75] and UniProt [73]. The ‘coordinations’ of the amino acid sequence in the PDB files were adjusted to match the ‘coordination’ of dbSNP. The distance between the polymorphism site and the ligand binding pocket of the protein was visualized on PyMOL.

## Supporting Information

Figure S1 Workflow of construction and mining of the multiple antithesis chemical-protein interactome (CPI). (a) Determining the case (AGNL+) and control (AGNL−) drugs from FDA's adverse event reporting system and PubMed. (b) A visualization of the chemical-protein interactome. Proteins that are preferably interacted by case but not control drugs are highlighted in a red dashed rectangle, these being regarded as the candidates mediating CIA.
(TIF)

Figure S2 Structures of the 15 control molecules.
(TIF)

Figure S3 Similarity of protein binding profile between Clozapine and Olanzapine. (a). Ordered by positive PCC value, the four CLZ-OLZ pairs ranked at the top 0.86, 2.51, 16.60 and 17.15 percentile of all possible pairs among 255 drug molecules, respectively. (b) The background distribution of the mean PCC of the four drug molecules were generated by randomly recruiting 10,000 sets with four drug pairs among all 255 drugs. CLZ and OLZ have highly similar protein binding profiles in terms of significantly high PCC of Z′-score vectors.
(TIF)

Table S1 The 410 protein pockets and their enzyme commission number.
(DOC)

Table S2 Drug molecules involved in CPI.
(DOC)

Table S3 ANOVA of the chemical-protein interactive effect before and after 2-directional Z-transformation.
(DOC)
