# Supplementary figures and images for: Exploring Off-Targets and Off-Systems for Adverse Drug Reactions via Chemical-Protein Interactome — Clozapine-Induced Agranulocytosis as a Case Study
Source: PLoS Comput Biol. 2011 Mar 31;7(3):e1002016. doi: 10.1371/journal.pcbi.1002016 (PMC3068927; doi:10.1371/journal.pcbi.1002016)

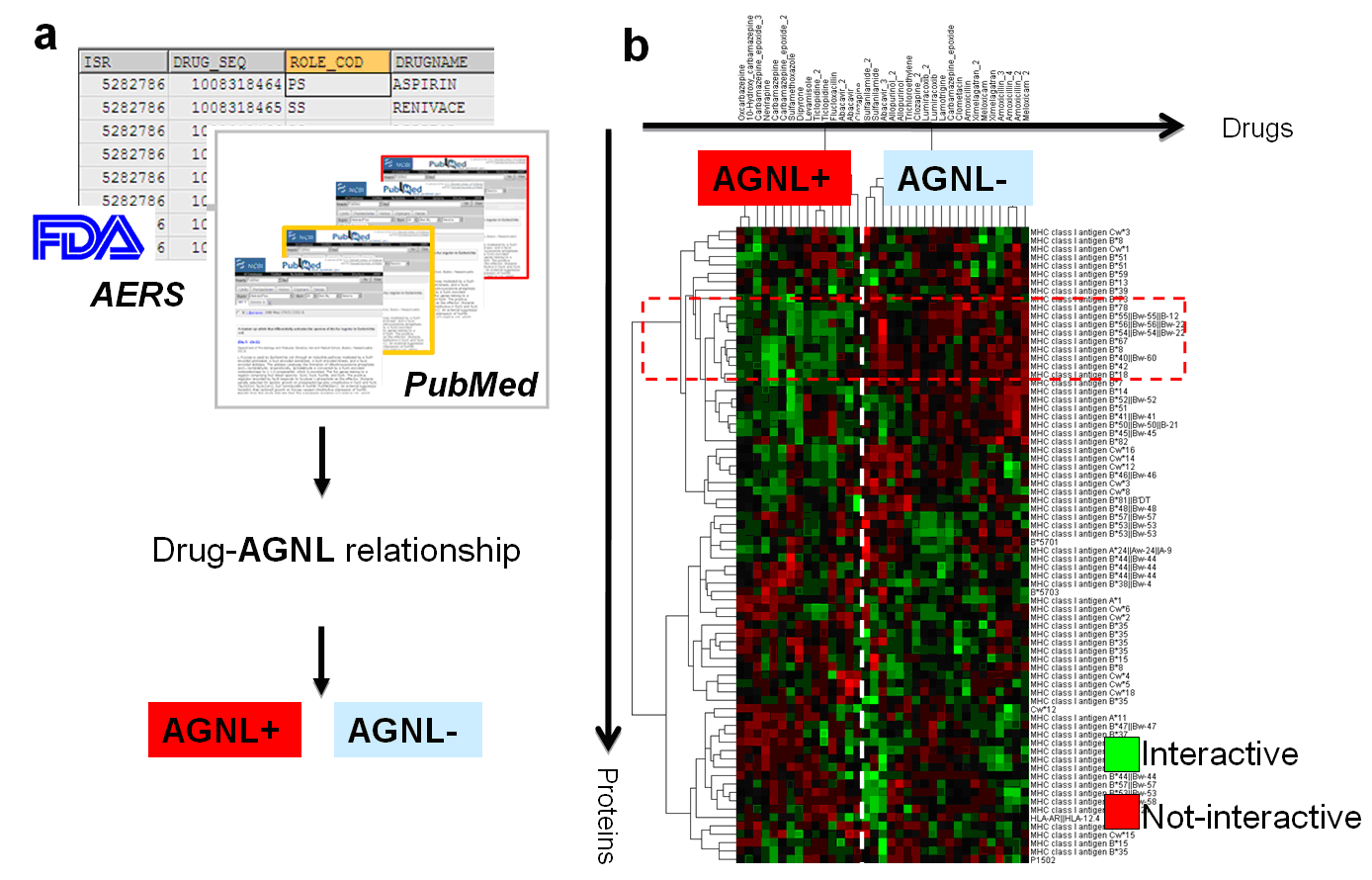

Supplement: Figure S1 — Workflow of construction and mining of the multiple antithesis chemical-protein interactome (CPI). (a) Determining the case (AGNL+) and control (AGNL−) drugs from FDA's adverse event reporting system and PubMed. (b) A visualization of the chemical-protein interactome. Proteins that are preferably interacted by case but not control drugs are highlighted in a red dashed rectangle, these being regarded as the candidates mediating CIA. (TIF) [file pcbi.1002016.s001.tif]

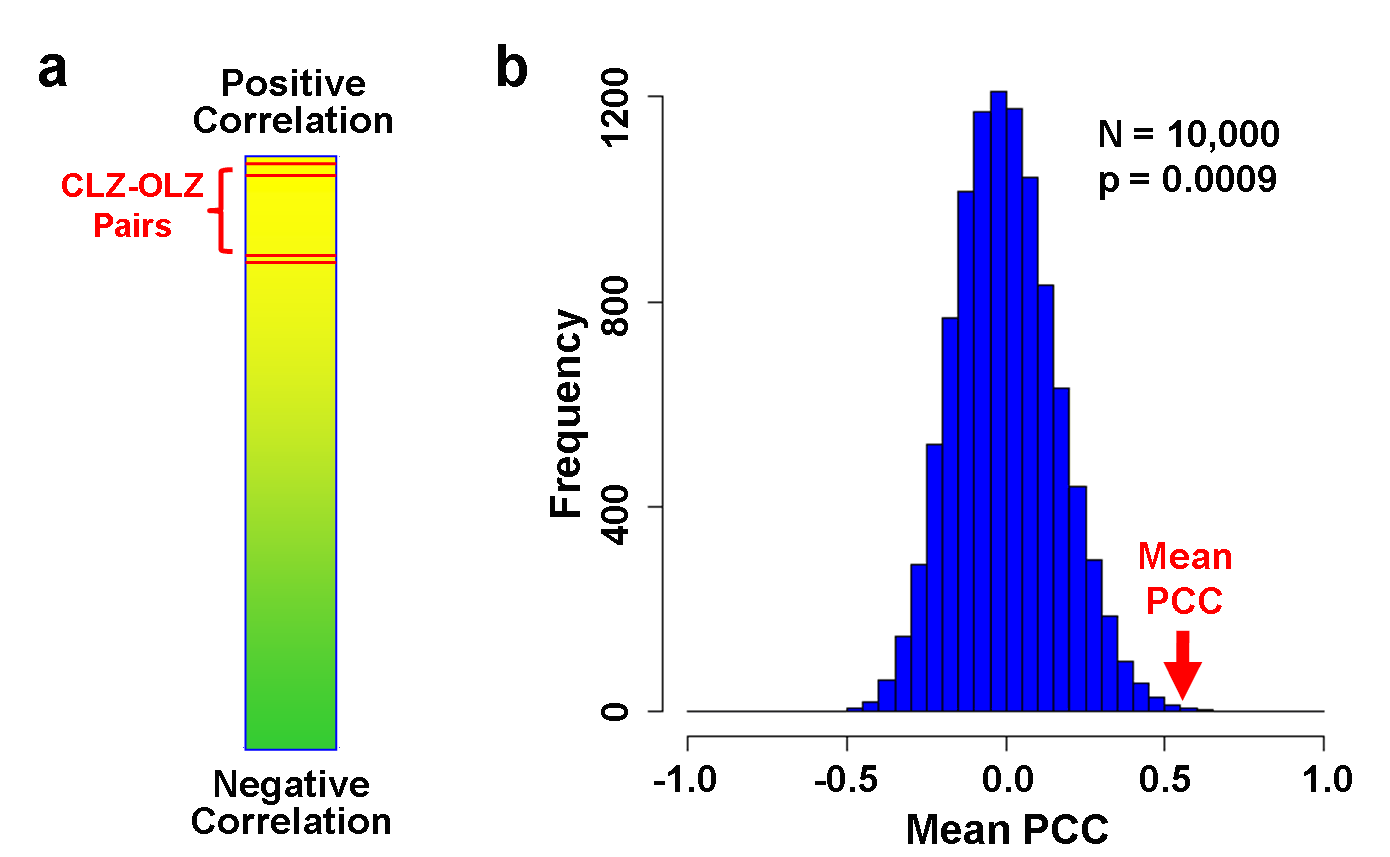

Supplement: Figure S3 — Similarity of protein binding profile between Clozapine and Olanzapine. (a). Ordered by positive PCC value, the four CLZ-OLZ pairs ranked at the top 0.86, 2.51, 16.60 and 17.15 percentile of all possible pairs among 255 drug molecules, respectively. (b) The background distribution of the mean PCC of the four drug molecules were generated by randomly recruiting 10,000 sets with four drug pairs among all 255 drugs. CLZ and OLZ have highly similar protein binding profiles in terms of significantly high PCC of Z′-score vectors. (TIF) [file pcbi.1002016.s003.tif]
