# Supplementary material for: Exploring Off-Targets and Off-Systems for Adverse Drug Reactions via Chemical-Protein Interactome — Clozapine-Induced Agranulocytosis as a Case Study
Source: PLoS Comput Biol. 2011 Mar 31;7(3):e1002016. doi: 10.1371/journal.pcbi.1002016 (PMC3068927; doi:10.1371/journal.pcbi.1002016)
Supplement: Table S1 — The 410 protein pockets and their enzyme commission number. (DOC) [file pcbi.1002016.s004.doc]

Table S1. The 410 protein pockets and their enzyme commission number

| PDB ID # | Target Name | EC Number * |
| --- | --- | --- |
| 11GS | Glutathione S-transferase P | 2.5.1.18 |
| 1A0L | Tryptase beta-2 | 3.4.21.59 |
| 1A2B | Transforming protein RhoA |  |
| 1A3B | Prothrombin | 3.4.21.5 |
| 1A42 | Carbonic anhydrase 2 | 4.2.1.1 |
| 1A5Y | Tyrosine-protein phosphatase non-receptor type 1 | 3.1.3.48 |
| 1A7A_1 | Adenosylhomocysteinase | 3.3.1.1 |
| 1A7A_2 | Adenosylhomocysteinase | 3.3.1.1 |
| 1A7C_1 | Plasminogen activator inhibitor 1 |  |
| 1A7C_2 | Plasminogen activator inhibitor 1 |  |
| 1A8M | Tumor necrosis factor |  |
| 1A9U | Mitogen-activated protein kinase 14 | 2.7.11.24 |
| 1AD5 | Tyrosine-protein kinase HCK | 2.7.10.2 |
| 1ANG | Angiogenin | 3.1.27.- |
| 1ATK | Cathepsin K | 3.4.22.38 |
| 1AUT | Vitamin K-dependent protein C | 3.4.21.69 |
| 1AXN | Annexin A3 |  |
| 1B09 | C-reactive protein |  |
| 1B1C | NADPH--cytochrome P450 reductase | 1.6.2.4 |
| 1B2T | Fractalkine |  |
| 1B56 | Fatty acid-binding protein, epidermal |  |
| 1B6A | Methionine aminopeptidase 2 | 3.4.11.18 |
| 1BIO | Complement factor D | 3.4.21.46 |
| 1BJ4 | Serine hydroxymethyltransferase, cytosolic | 2.1.2.1 |
| 1BKC | Disintegrin and metalloproteinase domain-containing protein 17 | 3.4.24.86 |
| 1BOZ | Dihydrofolate reductase | 1.5.1.3 |
| 1BP1 | Bactericidal permeability-increasing protein |  |
| 1BQS | Mucosal addressin cell adhesion molecule 1 |  |
| 1BX4 | Adenosine kinase | 2.7.1.20 |
| 1BYG | Tyrosine-protein kinase CSK | 2.7.10.2 |
| 1BZY | Hypoxanthine-guanine phosphoribosyltransferase | 2.4.2.8 |
| 1C1Y | Ras-related protein Rap-1A |  |
| 1C8P | Cytokine receptor common subunit beta |  |
| 1C9H | Peptidyl-prolyl cis-trans isomerase FKBP1B | 5.2.1.8 |
| 1CBS | Cellular retinoic acid-binding protein 2 |  |
| 1CGH | Cathepsin G | 3.4.21.20 |
| 1CM8 | Mitogen-activated protein kinase 12 | 2.7.11.24 |
| 1CSB | Cathepsin B | 3.4.22.1 |
| 1CTR | Calmodulin |  |
| 1CVI | Prostatic acid phosphatase | 3.1.3.2 |
| 1CYN | Peptidyl-prolyl cis-trans isomerase B | 5.2.1.8 |
| 1D0A | TNF receptor-associated factor 2 |  |
| 1D1T | Alcohol dehydrogenase class 4 mu/sigma chain | 1.1.1.1 |
| 1D2V | Myeloperoxidase | 1.11.1.7 |
| 1D3H_1 | Dihydroorotate dehydrogenase, mitochondrial | 1.3.5.2 |
| 1D3H_2 | Dihydroorotate dehydrogenase, mitochondrial | 1.3.5.2 |
| 1D4W | SH2 domain-containing protein 1A |  |
| 1D5R | Phosphatidylinositol-3,4,5-trisphosphate 3-phosphatase and dual-specificity protein phosphatase PTEN | 3.1.3.67||3.1.3.16||3.1.3.48 |
| 1D7K | Ornithine decarboxylase | 4.1.1.17 |
| 1DB1 | Vitamin D3 receptor |  |
| 1DB4 | Phospholipase A2, membrane associated | 3.1.1.4 |
| 1DFV | Neutrophil gelatinase-associated lipocalin |  |
| 1DHT | Estradiol 17-beta-dehydrogenase 1 | 1.1.1.62 |
| 1DIA | Formyltetrahydrofolate synthetase | 1.5.1.5||3.5.4.9||6.3.4.3 |
| 1DKF | Retinoic acid receptor alpha |  |
| 1DMT | Neprilysin | 3.4.24.11 |
| 1DS6 | Ras-related C3 botulinum toxin substrate 2 |  |
| 1E2D | Thymidylate kinase | 2.7.4.9 |
| 1E3G | Androgen receptor |  |
| 1E51 | Delta-aminolevulinic acid dehydratase | 4.2.1.24 |
| 1E8Z | Phosphatidylinositol-4,5-bisphosphate 3-kinase catalytic subunit gamma isoform | 2.7.1.153 |
| 1E96 | Ras-related C3 botulinum toxin substrate 1 |  |
| 1EA6 | Mismatch repair endonuclease PMS2 | 3.1.-.- |
| 1EAX | Suppressor of tumorigenicity protein 14 | 3.4.21.109 |
| 1EEM | Glutathione S-transferase omega-1 | 2.5.1.18 |
| 1EH8 | Methylated-DNA--protein-cysteine methyltransferase | 2.1.1.63 |
| 1EK5 | UDP-glucose 4-epimerase | 5.1.3.2 |
| 1ELV | Complement C1s subcomponent | 3.4.21.42 |
| 1ERU | Thioredoxin |  |
| 1ES7 | Bone morphogenetic protein 2 |  |
| 1ETA | Transthyretin |  |
| 1EXX | Retinoic acid receptor gamma |  |
| 1EZF | Squalene synthetase | 2.5.1.21 |
| 1F0R | Coagulation factor X | 3.4.21.6 |
| 1F0Y | Hydroxyacyl-coenzyme A dehydrogenase, mitochondrial | 1.1.1.35 |
| 1F2Q | High affinity immunoglobulin epsilon receptor subunit alpha |  |
| 1F3M | Serine/threonine-protein kinase PAK 1 | 2.7.11.1 |
| 1F5F | Sex hormone-binding globulin |  |
| 1F5N | Interferon-induced guanylate-binding protein 1 |  |
| 1F6O | DNA-3-methyladenine glycosylase | 3.2.2.21 |
| 1F8U | Acetylcholinesterase | 3.1.1.7 |
| 1FBY | Retinoic acid receptor RXR-alpha |  |
| 1FE3 | Fatty acid-binding protein, brain |  |
| 1FGG | Galactosylgalactosylxylosylprotein 3-beta-glucuronosyltransferase 3 | 2.4.1.135 |
| 1FIE | Coagulation factor XIII A chain | 2.3.2.13 |
| 1FO2 | Endoplasmic reticulum mannosyl-oligosaccharide 1,2-alpha-mannosidase | 3.2.1.113 |
| 1FPR | Tyrosine-protein phosphatase non-receptor type 6 | 3.1.3.48 |
| 1FQ1 | Cyclin-dependent kinase inhibitor 3 | 3.1.3.48||3.1.3.16 |
| 1FT4 | Tumor necrosis factor receptor superfamily member 1A |  |
| 1FTA | Fructose-1,6-bisphosphatase 1 | 3.1.3.11 |
| 1FW1 | Maleylacetoacetate isomerase | 5.2.1.2||2.5.1.18 |
| 1FYN | Proto-oncogene tyrosine-protein kinase Fyn | 2.7.10.2 |
| 1G0X | Leukocyte immunoglobulin-like receptor subfamily B member 1 |  |
| 1G0Y | Interleukin-1 receptor type I |  |
| 1G1T | E-selectin |  |
| 1G3M_1 | Estrogen sulfotransferase | 2.8.2.4 |
| 1G3M_2 | Estrogen sulfotransferase | 2.8.2.4 |
| 1G47 | LIM and senescent cell antigen-like-containing domain protein 1 |  |
| 1G54 | Carbonic anhydrase 4 | 4.2.1.1 |
| 1G55 | tRNA (cytosine-5-)-methyltransferase | 2.1.1.29 |
| 1G8I | Neuronal calcium sensor 1 |  |
| 1G8Q | CD81 antigen |  |
| 1GCZ | Macrophage migration inhibitory factor | 5.3.2.1||5.3.3.12 |
| 1GFW | Caspase-3 | 3.4.22.56 |
| 1GMN | Hepatocyte growth factor |  |
| 1GOS_1 | Amine oxidase [flavin-containing] B | 1.4.3.4 |
| 1GOS_2 | Amine oxidase [flavin-containing] B | 1.4.3.4 |
| 1GRE | Glutathione reductase, mitochondrial | 1.8.1.7 |
| 1GZP | T-cell surface glycoprotein CD1b |  |
| 1GZU | Nicotinamide mononucleotide adenylyltransferase 1 | 2.7.7.1||2.7.7.18 |
| 1H0C | Serine--pyruvate aminotransferase | 2.6.1.51||2.6.1.44 |
| 1H1B | Leukocyte elastase | 3.4.21.37 |
| 1H2K | Hypoxia-inducible factor 1-alpha inhibitor | 1.14.11.16 |
| 1H4W | Trypsin-3 | 3.4.21.4 |
| 1H9Z | Serum albumin |  |
| 1HA2 | Serum albumin |  |
| 1HAK | Annexin A5 |  |
| 1HDR | Dihydropteridine reductase | 1.5.1.34 |
| 1HE5_1 | Flavin reductase | 1.5.1.30||1.3.1.24 |
| 1HE5_2 | Flavin reductase | 1.5.1.30||1.3.1.24 |
| 1HFC | Interstitial collagenase | 3.4.24.7 |
| 1HKI | Chitotriosidase-1 | 3.2.1.14 |
| 1HMR | Fatty acid-binding protein, heart |  |
| 1HNF_1 | T-cell surface antigen CD2 |  |
| 1HNF_2 | T-cell surface antigen CD2 |  |
| 1HP7_1 | Alpha-1-antitrypsin |  |
| 1HP7_2 | Alpha-1-antitrypsin |  |
| 1HRK | Ferrochelatase, mitochondrial | 4.99.1.1 |
| 1HS6 | Leukotriene A-4 hydrolase | 3.3.2.6 |
| 1HSO | Alcohol dehydrogenase 1A | 1.1.1.1 |
| 1HSZ | Alcohol dehydrogenase 1B | 1.1.1.1 |
| 1HT0 | Alcohol dehydrogenase 1C | 1.1.1.1 |
| 1HTI | Triosephosphate isomerase | 5.3.1.1 |
| 1HUR | ADP-ribosylation factor 1 |  |
| 1HWL | 3-hydroxy-3-methylglutaryl-coenzyme A reductase | 1.1.1.34 |
| 1HYI | Early endosome antigen 1 |  |
| 1I0Z | L-lactate dehydrogenase B chain | 1.1.1.27 |
| 1I10 | L-lactate dehydrogenase A chain | 1.1.1.27 |
| 1I1N | Protein-L-isoaspartate(D-aspartate) O-methyltransferase | 2.1.1.77 |
| 1I71 | Apolipoprotein(a) | 3.4.21.- |
| 1I7B | S-adenosylmethionine decarboxylase proenzyme | 4.1.1.50 |
| 1I7I | Peroxisome proliferator-activated receptor gamma |  |
| 1I92 | Ezrin-radixin-moesin-binding phosphoprotein 50 |  |
| 1ICE | Caspase-1 | 3.4.22.36 |
| 1IH0 | Troponin C, slow skeletal and cardiac muscles |  |
| 1IHI_1 | Aldo-keto reductase family 1 member C2 | 1.-.-.-||1.3.1.20||1.1.1.213 |
| 1IHI_2 | Aldo-keto reductase family 1 member C2 | 1.-.-.-||1.3.1.20||1.1.1.213 |
| 1IMB | Inositol monophosphatase | 3.1.3.25 |
| 1IRJ | Protein S100-A9 |  |
| 1ITU | Dipeptidase 1 | 3.4.13.19 |
| 1IYH | Glutathione-requiring prostaglandin D synthase | 5.3.99.2||2.5.1.18 |
| 1J1B | Glycogen synthase kinase-3 beta | 2.7.11.26 |
| 1J4I | Peptidyl-prolyl cis-trans isomerase FKBP1A | 5.2.1.8 |
| 1J78 | Vitamin D-binding protein |  |
| 1J8F | NAD-dependent deacetylase sirtuin-2 | 3.5.1.- |
| 1J99 | Bile salt sulfotransferase | 2.8.2.14 |
| 1JAP | Neutrophil collagenase | 3.4.24.34 |
| 1JBQ | Cystathionine beta-synthase | 4.2.1.22 |
| 1JCN | Inosine-5'-monophosphate dehydrogenase 1 | 1.1.1.205 |
| 1JD0 | Carbonic anhydrase 12 | 4.2.1.1 |
| 1JKL | Death-associated protein kinase 1 | 2.7.11.1 |
| 1JNK | Mitogen-activated protein kinase 10 | 2.7.11.24 |
| 1JUJ_1 | Thymidylate synthase | 2.1.1.45 |
| 1JUJ_2 | Thymidylate synthase | 2.1.1.45 |
| 1JWH | Casein kinase II subunit alpha | 2.7.11.1 |
| 1K3Y | Glutathione S-transferase A1 | 2.5.1.18 |
| 1K7L | Peroxisome proliferator-activated receptor alpha |  |
| 1K86 | Caspase-7 | 3.4.22.60 |
| 1KBQ | NAD(P)H dehydrogenase [quinone] 1 | 1.6.5.2 |
| 1KJL | Galectin-3 |  |
| 1KPF | Histidine triad nucleotide-binding protein 1 | 3.-.-.- |
| 1KTA | Branched-chain-amino-acid aminotransferase, mitochondrial | 2.6.1.42 |
| 1L7X_1 | Glycogen phosphorylase, liver form | 2.4.1.1 |
| 1L7X_2 | Glycogen phosphorylase, liver form | 2.4.1.1 |
| 1LCL | Eosinophil lysophospholipase | 3.1.1.5 |
| 1LJR | Glutathione S-transferase theta-2 | 2.5.1.18 |
| 1LQV | Endothelial protein C receptor |  |
| 1LT8 | Betaine--homocysteine S-methyltransferase 1 | 2.1.1.5 |
| 1LYW | Cathepsin D | 3.4.23.5 |
| 1M17 | Epidermal growth factor receptor | 2.7.10.1 |
| 1M4U | Bone morphogenetic protein 7 |  |
| 1MC5_1 | Alcohol dehydrogenase class-3 | 1.1.1.1||1.1.1.284||1.1.1.- |
| 1MC5_2 | Alcohol dehydrogenase class-3 | 1.1.1.1||1.1.1.284||1.1.1.- |
| 1MLW | Tryptophan 5-hydroxylase 1 | 1.14.16.4 |
| 1MMQ | Matrilysin | 3.4.24.23 |
| 1MQ0 | Cytidine deaminase | 3.5.4.5 |
| 1MQB | Ephrin type-A receptor 2 | 2.7.10.1 |
| 1MRQ_1 | Aldo-keto reductase family 1 member C1 | 1.1.1.-||1.1.1.149||1.3.1.20||1.1.1.112 |
| 1MRQ_2 | Aldo-keto reductase family 1 member C1 | 1.1.1.-||1.1.1.149||1.3.1.20||1.1.1.112 |
| 1MUO | Serine/threonine-protein kinase 6 | 2.7.11.1 |
| 1N7I | Phenylethanolamine N-methyltransferase | 2.1.1.28 |
| 1NAV | Thyroid hormone receptor alpha |  |
| 1NFB_1 | Inosine-5'-monophosphate dehydrogenase 2 | 1.1.1.205 |
| 1NFB_2 | Inosine-5'-monophosphate dehydrogenase 2 | 1.1.1.205 |
| 1NHZ | Glucocorticoid receptor |  |
| 1NI4 | Pyruvate dehydrogenase E1 component subunit beta, mitochondrial | 1.2.4.1 |
| 1NM8 | Carnitine O-acetyltransferase | 2.3.1.7 |
| 1NN6 | Chymase | 3.4.21.39 |
| 1NRG | Pyridoxine-5'-phosphate oxidase | 1.4.3.5 |
| 1NRL | Nuclear receptor subfamily 1 group I member 2 |  |
| 1NSI | Nitric oxide synthase, inducible | 1.14.13.39 |
| 1NY3 | MAP kinase-activated protein kinase 2 | 2.7.11.1 |
| 1O6L | RAC-beta serine/threonine-protein kinase | 2.7.11.1 |
| 1OAT | Ornithine aminotransferase, mitochondrial | 2.6.1.13 |
| 1OF7 | Aldehyde dehydrogenase, mitochondrial | 1.2.1.3 |
| 1OGS | Glucosylceramidase | 3.2.1.45 |
| 1OIQ | Cell division protein kinase 2 | 2.7.11.22 |
| 1OIZ | Alpha-tocopherol transfer protein |  |
| 1OLM | SEC14-like protein 2 |  |
| 1OLS | 2-oxoisovalerate dehydrogenase subunit alpha, mitochondrial | 1.2.4.4 |
| 1ONQ | T-cell surface glycoprotein CD1a |  |
| 1OPL | Proto-oncogene tyrosine-protein kinase ABL1 | 2.7.10.2 |
| 1ORE | Adenine phosphoribosyltransferase | 2.4.2.7 |
| 1OSH | Bile acid receptor |  |
| 1OTH | Ornithine carbamoyltransferase, mitochondrial | 2.1.3.3 |
| 1P49 | Steryl-sulfatase | 3.1.6.2 |
| 1P4M_1 | Riboflavin kinase | 2.7.1.26 |
| 1P4M_2 | Riboflavin kinase | 2.7.1.26 |
| 1P4R | Bifunctional purine biosynthesis protein PURH | 2.1.2.3||3.5.4.10 |
| 1P5J | L-serine dehydratase | 4.3.1.17||4.3.1.19 |
| 1PIC | Phosphatidylinositol 3-kinase regulatory subunit alpha |  |
| 1PIN | Peptidyl-prolyl cis-trans isomerase NIMA-interacting 1 | 5.2.1.8 |
| 1PL7 | Sorbitol dehydrogenase | 1.1.1.14 |
| 1POZ | CD44 antigen |  |
| 1PQ2 | Cytochrome P450 2C8 | 1.14.14.1 |
| 1PSO | Pepsin A | 3.4.23.1 |
| 1PT9 | NAD(P) transhydrogenase, mitochondrial | 1.6.1.2 |
| 1PTW | cAMP-specific 3',5'-cyclic phosphodiesterase 4D | 3.1.4.17 |
| 1Q11 | Tyrosyl-tRNA synthetase, cytoplasmic | 6.1.1.1 |
| 1Q1Z | Sulfotransferase family cytosolic 2B member 1 | 2.8.2.2 |
| 1Q4O | Serine/threonine-protein kinase PLK1 | 2.7.11.21 |
| 1QAB | Retinol-binding protein 4 |  |
| 1QCY | Integrin alpha-1 |  |
| 1QIA | Stromelysin-1 | 3.4.24.17 |
| 1QIP | Lactoylglutathione lyase | 4.4.1.5 |
| 1QJA | 14-3-3 protein zeta/delta |  |
| 1QMV | Peroxiredoxin-2 | 1.11.1.15 |
| 1QPC | Proto-oncogene tyrosine-protein kinase LCK | 2.7.10.2 |
| 1QR6 | NAD-dependent malic enzyme, mitochondrial | 1.1.1.38 |
| 1R47 | Alpha-galactosidase A | 3.2.1.22 |
| 1R4L | Angiotensin-converting enzyme 2 | 3.4.17.- |
| 1R55 | ADAM 33 | 3.4.24.- |
| 1R5K | Estrogen receptor |  |
| 1R6T | Tryptophanyl-tRNA synthetase, cytoplasmic | 6.1.1.2 |
| 1R74 | Glycine N-methyltransferase | 2.1.1.20 |
| 1R82 | Glycoprotein-fucosylgalactoside alpha-galactosyltransferase | 2.4.1.40||2.4.1.37 |
| 1R9O | Cytochrome P450 2C9 | 1.14.13.80||1.14.13.48||1.14.13.49 |
| 1RD4 | Integrin alpha-L |  |
| 1RFN | Coagulation factor IX | 3.4.21.22 |
| 1RT9 | Purine nucleoside phosphorylase | 2.4.2.1 |
| 1S8C | Heme oxygenase 1 | 1.14.99.3 |
| 1SD2 | S-methyl-5'-thioadenosine phosphorylase | 2.4.2.28 |
| 1SG0_1 | Ribosyldihydronicotinamide dehydrogenase [quinone] | 1.10.99.2 |
| 1SG0_2 | Ribosyldihydronicotinamide dehydrogenase [quinone] | 1.10.99.2 |
| 1SMO | Triggering receptor expressed on myeloid cells 1 |  |
| 1SQN | Progesterone receptor |  |
| 1T40_1 | Aldose reductase | 1.1.1.21 |
| 1T40_2 | Aldose reductase | 1.1.1.21 |
| 1TDI | Glutathione S-transferase A3 | 2.5.1.18 |
| 1TG2 | Phenylalanine-4-hydroxylase | 1.14.16.1 |
| 1TVO | Mitogen-activated protein kinase 1 | 2.7.11.24 |
| 1TYL | Insulin |  |
| 1U54 | Activated CDC42 kinase 1 | 2.7.10.2 |
| 1UDT | cGMP-specific 3',5'-cyclic phosphodiesterase | 3.1.4.35 |
| 1UHL_1 | Retinoic acid receptor RXR-beta |  |
| 1UHL_2 | Retinoic acid receptor RXR-beta |  |
| 1UKI | Mitogen-activated protein kinase 8 | 2.7.11.24 |
| 1UMK | NADH-cytochrome b5 reductase 3 | 1.6.2.2 |
| 1UOU | Thymidine phosphorylase | 2.4.2.4 |
| 1UWJ | B-Raf proto-oncogene serine/threonine-protein kinase | 2.7.11.1 |
| 1UZF | Angiotensin-converting enzyme | 3.4.15.1||3.2.1.- |
| 1V04 | Serum paraoxonase/arylesterase 1 | 3.1.1.2||3.1.8.1 |
| 1VCU | Sialidase-2 | 3.2.1.18 |
| 1VJ5 | Epoxide hydrolase 2 | 3.3.2.10 |
| 1VJA | Urokinase-type plasminogen activator | 3.4.21.73 |
| 1VJB | Estrogen-related receptor gamma |  |
| 1W0H | Histone mRNA 3'-exonuclease 1 | 3.1.-.- |
| 1W22 | Histone deacetylase 8 | 3.5.1.98 |
| 1W7N | Kynurenine--oxoglutarate transaminase 1 | 2.6.1.7||2.6.1.64||4.4.1.13 |
| 1WDA | Protein-arginine deiminase type-4 | 3.5.3.15 |
| 1WOK | Poly [ADP-ribose] polymerase 1 | 2.4.2.30 |
| 1WWA | High affinity nerve growth factor receptor | 2.7.10.1 |
| 1WWB | BDNF/NT-3 growth factors receptor | 2.7.10.1 |
| 1WWC | NT-3 growth factor receptor | 2.7.10.1 |
| 1X0O | Aryl hydrocarbon receptor nuclear translocator |  |
| 1X50 | Galectin-4 |  |
| 1XAP | Retinoic acid receptor beta |  |
| 1XBA | Tyrosine-protein kinase SYK | 2.7.10.2 |
| 1XF0_1 | Aldo-keto reductase family 1 member C3 | 1.-.-.-||1.3.1.20||1.1.1.213||1.1.1.63||1.1.1.64||1.1.1.188||1.1.1.112 |
| 1XF0_2 | Aldo-keto reductase family 1 member C3 | 1.-.-.-||1.3.1.20||1.1.1.213||1.1.1.63||1.1.1.64||1.1.1.188||1.1.1.112 |
| 1XLV | Cholinesterase | 3.1.1.8 |
| 1XMI | Cystic fibrosis transmembrane conductance regulator | 3.6.3.49 |
| 1XOS | cAMP-specific 3',5'-cyclic phosphodiesterase 4B | 3.1.4.17 |
| 1XQZ | Proto-oncogene serine/threonine-protein kinase Pim-1 | 2.7.11.1 |
| 1XU9_1 | Corticosteroid 11-beta-dehydrogenase isozyme 1 | 1.1.1.146 |
| 1XU9_2 | Corticosteroid 11-beta-dehydrogenase isozyme 1 | 1.1.1.146 |
| 1XVP | Nuclear receptor subfamily 1 group I member 3 |  |
| 1XWK | Glutathione S-transferase Mu 1 | 2.5.1.18 |
| 1Y0S | Peroxisome proliferator-activated receptor delta |  |
| 1Y0X | Thyroid hormone receptor beta |  |
| 1YB5 | Quinone oxidoreductase | 1.6.5.5 |
| 1YET | Heat shock protein HSP 90-alpha |  |
| 1YOL | Proto-oncogene tyrosine-protein kinase Src | 2.7.10.2 |
| 1YV5 | Farnesyl pyrophosphate synthetase | 2.5.1.1||2.5.1.10 |
| 1YVJ | Tyrosine-protein kinase JAK3 | 2.7.10.2 |
| 1Z57 | Dual specificity protein kinase CLK1 | 2.7.12.1 |
| 1Z6J | Coagulation factor VII | 3.4.21.21 |
| 1Z6T | Apoptotic protease-activating factor 1 |  |
| 1Z8D_1 | Glycogen phosphorylase, muscle form | 2.4.1.1 |
| 1Z8D_2 | Glycogen phosphorylase, muscle form | 2.4.1.1 |
| 1Z8G | Serine protease hepsin | 3.4.21.106 |
| 1Z93 | Carbonic anhydrase 3 | 4.2.1.1 |
| 1ZBQ | 3-alpha,7-alpha,12-alpha-trihydroxy-5-beta-cholest-24-enoyl-CoA hydratase | 1.1.1.35||4.2.1.107 |
| 1ZJK | Mannan-binding lectin serine protease 2 | 3.4.21.104 |
| 1ZKK_1 | Histone-lysine N-methyltransferase SETD8 | 2.1.1.43 |
| 1ZKK_2 | Histone-lysine N-methyltransferase SETD8 | 2.1.1.43 |
| 1ZNQ | Glyceraldehyde-3-phosphate dehydrogenase | 1.2.1.12 |
| 1ZSX | Voltage-gated potassium channel subunit beta-2 |  |
| 1ZT3 | Insulin-like growth factor-binding protein 1 |  |
| 1ZV4 | Regulator of G-protein signaling 17 |  |
| 1ZX0 | Guanidinoacetate N-methyltransferase | 2.1.1.2 |
| 1ZXM | DNA topoisomerase 2-alpha | 5.99.1.3 |
| 1ZXQ | Intercellular adhesion molecule 2 |  |
| 2A3I | Mineralocorticoid receptor |  |
| 2AB6 | Glutathione S-transferase Mu 2 | 2.5.1.18 |
| 2AC3 | MAP kinase-interacting serine/threonine-protein kinase 2 | 2.7.11.1 |
| 2AEB | Arginase-1 | 3.5.3.1 |
| 2AHE | Chloride intracellular channel protein 4 |  |
| 2ANY | Plasma kallikrein | 3.4.21.34 |
| 2AOU | Histamine N-methyltransferase | 2.1.1.8 |
| 2AUH | Insulin receptor | 2.7.10.1 |
| 2AVD | Catechol-O-methyltransferase domain-containing protein 1 | 2.1.1.- |
| 2AYO | Ubiquitin carboxyl-terminal hydrolase 14 | 3.1.2.15 |
| 2B3K | Methionine aminopeptidase 1 | 3.4.11.18 |
| 2B4Y_1 | NAD-dependent deacetylase sirtuin-5 | 3.5.1.- |
| 2B4Y_2 | NAD-dependent deacetylase sirtuin-5 | 3.5.1.- |
| 2B7A | Tyrosine-protein kinase JAK2 | 2.7.10.2 |
| 2BH9 | Glucose-6-phosphate 1-dehydrogenase | 1.1.1.49 |
| 2BIY | 3-phosphoinositide-dependent protein kinase 1 | 2.7.11.1 |
| 2BRO | Serine/threonine-protein kinase Chk1 | 2.7.11.1 |
| 2BU5 | [Pyruvate dehydrogenase [lipoamide]] kinase isozyme 2, mitochondrial | 2.7.11.2 |
| 2BX8_1 | Serum albumin |  |
| 2BX8_2 | Serum albumin |  |
| 2BX8_3 | Serum albumin |  |
| 2BXF | Serum albumin |  |
| 2C2Z | Caspase-8 | 3.4.22.61 |
| 2C30 | Serine/threonine-protein kinase PAK 6 | 2.7.11.1 |
| 2C3Q | Glutathione S-transferase theta-1 | 2.5.1.18 |
| 2C47 | Casein kinase I isoform gamma-2 | 2.7.11.1 |
| 2C6C | Glutamate carboxypeptidase 2 | 3.4.17.21 |
| 2C6Q | GMP reductase 2 | 1.7.1.7 |
| 2C9V | Superoxide dismutase [Cu-Zn] | 1.15.1.1 |
| 2CAB | Carbonic anhydrase 1 | 4.2.1.1 |
| 2CFI | 10-formyltetrahydrofolate dehydrogenase | 1.5.1.6 |
| 2CFY | Thioredoxin reductase 1, cytoplasmic | 1.8.1.9 |
| 2CG5 | L-aminoadipate-semialdehyde dehydrogenase-phosphopantetheinyl transferase | 2.7.8.- |
| 2CKG | Sentrin-specific protease 1 | 3.4.22.- |
| 2CYK | Interleukin-4 |  |
| 2CZH | Inositol monophosphatase 2 | 3.1.3.25 |
| 2DFD | Malate dehydrogenase, mitochondrial | 1.1.1.37 |
| 2E8A | Heat shock 70 kDa protein 1 |  |
| 2EU9 | Dual specificity protein kinase CLK3 | 2.7.12.1 |
| 2F2S | Acetyl-CoA acetyltransferase, mitochondrial | 2.3.1.9 |
| 2F57 | Serine/threonine-protein kinase PAK 7 | 2.7.11.1 |
| 2F9Q | Cytochrome P450 2D6 | 1.14.14.1 |
| 2FGI | Basic fibroblast growth factor receptor 1 | 2.7.10.1 |
| 2FKY | Kinesin-like protein KIF11 |  |
| 2FOJ | Ubiquitin carboxyl-terminal hydrolase 7 | 3.1.2.15 |
| 2FYB | Beta-1,4-galactosyltransferase 1 | 2.4.1.-||2.4.1.22||2.4.1.90||2.4.1.38 |
| 2G1N | Renin | 3.4.23.15 |
| 2GK1 | Beta-hexosaminidase subunit alpha | 3.2.1.52 |
| 2GL6 | Creatine kinase, sarcomeric mitochondrial | 2.7.3.2 |
| 2GLQ | Alkaline phosphatase, placental type | 3.1.3.1 |
| 2GU8 | cAMP-dependent protein kinase catalytic subunit alpha | 2.7.11.11 |
| 2H11 | Thiopurine S-methyltransferase | 2.1.1.67 |
| 2HGS_1 | Glutathione synthetase | 6.3.2.3 |
| 2HGS_2 | Glutathione synthetase | 6.3.2.3 |
| 2HHA | Dipeptidyl peptidase 4 | 3.4.14.5 |
| 2HI4 | Cytochrome P450 1A2 | 1.14.14.1 |
| 2HRB | Carbonyl reductase [NADPH] 3 | 1.1.1.184 |
| 2IIP | Nicotinamide N-methyltransferase | 2.1.1.1 |
| 2ILK | Interleukin-10 |  |
| 2J0D | Cytochrome P450 3A4 | 1.14.13.67||1.14.13.97||1.14.13.32 |
| 2J4E | Inosine triphosphate pyrophosphatase | 3.6.1.19 |
| 2NZ2 | Argininosuccinate synthase | 6.3.4.5 |
| 2O05 | Spermidine synthase | 2.5.1.16 |
| 2O23 | 3-hydroxyacyl-CoA dehydrogenase type-2 | 1.1.1.35||1.1.1.178 |
| 2OJ9 | Insulin-like growth factor 1 receptor | 2.7.10.1 |
| 2ORV | Thymidine kinase, cytosolic | 2.7.1.21 |
| 2PFR | Arylamine N-acetyltransferase 2 | 2.3.1.5 |
| 2PK4 | Plasminogen | 3.4.21.7 |
| 2PVY | Fibroblast growth factor receptor 2 | 2.7.10.1 |
| 2QY0 | Complement C1r subcomponent | 3.4.21.41 |
| 2QYK | cAMP-specific 3',5'-cyclic phosphodiesterase 4A | 3.1.4.17 |
| 2RCT | Retinol-binding protein 2 |  |
| 2VGB | Pyruvate kinase isozymes R/L | 2.7.1.40 |
| 2VQM | Histone deacetylase 4 | 3.5.1.98 |
| 2Z5F | Sulfotransferase family cytosolic 1B member 1 | 2.8.2.- |
| 2Z5X | Amine oxidase [flavin-containing] A | 1.4.3.4 |
| 2Z7R | Ribosomal protein S6 kinase alpha-1 | 2.7.11.1 |
| 3BWY | Catechol O-methyltransferase | 2.1.1.6 |
| 3C0Z | Histone deacetylase 7 | 3.5.1.98 |
| 3CQW | RAC-alpha serine/threonine-protein kinase | 2.7.11.1 |
| 3DDU | Prolyl endopeptidase | 3.4.21.26 |
| 3DRB | Creatine kinase B-type | 2.7.3.2 |
| 3DYD | Tyrosine aminotransferase | 2.6.1.5 |
| 3JDW | Glycine amidinotransferase, mitochondrial | 2.1.4.1 |
| 4GTU | Glutathione S-transferase Mu 4 | 2.5.1.18 |
| 5GAL | Galectin-7 |  |
| 5P21 | GTPase HRas |  |

^#^ An entry name that is ended with a number represents the pocket number of a certain PDB structure.

* EC numbers begin with 1 denotes the oxidoreductases.
