# Supplementary material for: Exploring Off-Targets and Off-Systems for Adverse Drug Reactions via Chemical-Protein Interactome — Clozapine-Induced Agranulocytosis as a Case Study
Source: PLoS Comput Biol. 2011 Mar 31;7(3):e1002016. doi: 10.1371/journal.pcbi.1002016 (PMC3068927; doi:10.1371/journal.pcbi.1002016)
Supplement: Table S2 — Drug molecules involved in CPI. (DOC) [file pcbi.1002016.s005.doc]

Table S2. Drug molecules involved in CPI.

| Drugname# | Class | If related* |
| --- | --- | --- |
| Acetaminophen | Agranulocytosis | 1 |
| Allopurinol | Agranulocytosis | 1 |
| Allopurinol 2 | Agranulocytosis | 1 |
| Captopril | Agranulocytosis | 1 |
| Carbamazepine | Agranulocytosis | 1 |
| Carbamazepine 2 | Agranulocytosis | 1 |
| Cefuroxime | Agranulocytosis | 1 |
| Chlorprothixene | Agranulocytosis | 0 |
| Clindamycin | Agranulocytosis | 1 |
| Clozapine | Agranulocytosis | 1 |
| Clozapine 2 | Agranulocytosis | 1 |
| Dapsone | Agranulocytosis | 1 |
| Debrisoquin | Agranulocytosis | 0 |
| Dexfenfluramine | Agranulocytosis | 0 |
| Diclofenac | Agranulocytosis | 1 |
| Doxycycline | Agranulocytosis | 1 |
| Doxycycline 2 | Agranulocytosis | 1 |
| Fenoterol | Agranulocytosis | 0 |
| Grepafloxacin | Agranulocytosis | 0 |
| Guanethidine | Agranulocytosis | 0 |
| Haloperidol | Agranulocytosis | 1 |
| Ibuprofen | Agranulocytosis | 1 |
| Ibuprofen 2 | Agranulocytosis | 1 |
| Ibuprofen 3 | Agranulocytosis | 1 |
| Ibuprofen 4 | Agranulocytosis | 1 |
| Isocarboxazid | Agranulocytosis | 0 |
| Naproxen | Agranulocytosis | 1 |
| Nifedipine | Agranulocytosis | 1 |
| Nitrofurantoin | Agranulocytosis | 1 |
| Olanzapine | Agranulocytosis | 0 |
| Olanzapine 2 | Agranulocytosis | 0 |
| Omeprazole | Agranulocytosis | 1 |
| Omeprazole 2 | Agranulocytosis | 1 |
| Omeprazole 3 | Agranulocytosis | 1 |
| Omeprazole 4 | Agranulocytosis | 1 |
| Phenelzine | Agranulocytosis | 0 |
| Phenytoin | Agranulocytosis | 1 |
| Phenytoin 2 | Agranulocytosis | 1 |
| Pipobroman | Agranulocytosis | 0 |
| Propericiazine | Agranulocytosis | 0 |
| Ranitidine | Agranulocytosis | 1 |
| Spironolactone | Agranulocytosis | 1 |
| Sulfasalazine | Agranulocytosis | 1 |
| Sulfasalazine 2 | Agranulocytosis | 1 |
| Sulfasalazine 3 | Agranulocytosis | 1 |
| Sulfasalazine 4 | Agranulocytosis | 1 |
| Terbutaline | Agranulocytosis | 0 |
| Terbutaline 2 | Agranulocytosis | 1 |
| Terbutaline 3 | Agranulocytosis | 1 |
| Terbutaline 4 | Agranulocytosis | 1 |
| Terbutaline 5 | Agranulocytosis | 1 |
| Terbutaline 6 | Agranulocytosis | 1 |
| Tinidazole | Agranulocytosis | 0 |
| Tridihexethyl | Agranulocytosis | 0 |
| Acetylcholine | Alzheimer | 0 |
| Amphetamine | Alzheimer | 0 |
| Amphetamine 4 | Alzheimer | 0 |
| Atorvastatin | Alzheimer | 0 |
| Azathioprine 2 | Alzheimer | 0 |
| Bexarotene | Alzheimer | 0 |
| Budesonide | Alzheimer | 0 |
| Captopril | Alzheimer | 0 |
| Celecoxib | Alzheimer | 0 |
| Cyproheptadine 2 | Alzheimer | 0 |
| Dexamethasone | Alzheimer | 0 |
| Donepezil 2 | Alzheimer | 1 |
| Erythromycin | Alzheimer | 0 |
| Ethambutol | Alzheimer | 0 |
| Ethambutol 2 | Alzheimer | 0 |
| Etonogestrel | Alzheimer | 0 |
| Fluticasone-propionate | Alzheimer | 0 |
| Furosemide | Alzheimer | 0 |
| Galantamine | Alzheimer | 1 |
| Galantamine 2 | Alzheimer | 1 |
| Gentamicin 2 | Alzheimer | 0 |
| Haloperidol | Alzheimer | 0 |
| Huperzine a | Alzheimer | 1 |
| Ibuprofen 4 | Alzheimer | 0 |
| Kanamycin 2 | Alzheimer | 0 |
| Levonorgestrel | Alzheimer | 0 |
| Lovastatin 2 | Alzheimer | 0 |
| Megestrol | Alzheimer | 0 |
| Memantine | Alzheimer | 1 |
| Memantine 2 | Alzheimer | 1 |
| Metronidazole | Alzheimer | 0 |
| Mometasone | Alzheimer | 0 |
| Netilmicin | Alzheimer | 0 |
| Omeprazole 4 | Alzheimer | 0 |
| Phenytoin 2 | Alzheimer | 0 |
| Physostigmine | Alzheimer | 1 |
| Physostigmine 2 | Alzheimer | 1 |
| Piroxicam | Alzheimer | 0 |
| Piroxicam 2 | Alzheimer | 0 |
| Rivastigmine | Alzheimer | 1 |
| Sildenafil | Alzheimer | 0 |
| Simvastatin | Alzheimer | 0 |
| Streptomycin | Alzheimer | 0 |
| Sulfasalazine 2 | Alzheimer | 0 |
| Sulfasalazine 4 | Alzheimer | 0 |
| Tacrine | Alzheimer | 1 |
| Tenoxicam | Alzheimer | 0 |
| Tenoxicam 2 | Alzheimer | 0 |
| Terbutaline 2 | Alzheimer | 0 |
| Ticlopidine | Alzheimer | 0 |
| Tobramycin 2 | Alzheimer | 0 |
| Tolbutamide | Alzheimer | 0 |
| Trichostatin a | Alzheimer | 0 |
| Valdecoxib | Alzheimer | 0 |
| Valproate | Alzheimer | 0 |
| Vardenafil | Alzheimer | 0 |
| Vorinostat | Alzheimer | 0 |
| 4,5-dianilinophthalimide | anti-Alzheimer drug | \N |
| Chlorpromazine | antipsychotics | \N |
| Clozapine | antipsychotics | \N |
| Clozapine 2 | antipsychotics | \N |
| Fluphenazine | antipsychotics | \N |
| Fluphenazine 2 | antipsychotics | \N |
| Haloperidol | antipsychotics | \N |
| Olanzapine 2 | antipsychotics | \N |
| Phenothiazine | antipsychotics | \N |
| Prochlorperazine | antipsychotics | \N |
| Prochlorperazine 2 | antipsychotics | \N |
| Thioridazine | antipsychotics | \N |
| Trifluoperazine | antipsychotics | \N |
| Trifluoperazine 2 | antipsychotics | \N |
| Amitriptyline | cholestasis | 1 |
| Amitriptyline 2 | cholestasis | 1 |
| Amoxicillin | cholestasis | 1 |
| Amoxicillin 2 | cholestasis | 1 |
| Ampicillin | cholestasis | 1 |
| Aprindine | cholestasis | 1 |
| Aspirin | cholestasis | 1 |
| Aspirin 2 | cholestasis | 1 |
| Azathioprine | cholestasis | 1 |
| Azathioprine 2 | cholestasis | 1 |
| Bumetanide | cholestasis | 0 |
| Captopril | cholestasis | 1 |
| Carbamazepine | cholestasis | 1 |
| Carbamazepine 2 | cholestasis | 1 |
| Celecoxib | cholestasis | 0 |
| Cephalexin | cholestasis | 0 |
| Cerivastatin | cholestasis | 0 |
| Chloramphenicol | cholestasis | 0 |
| Chlorothiazide | cholestasis | 1 |
| Chlorpromazine | cholestasis | 1 |
| Cyproheptadine | cholestasis | 1 |
| Cyproheptadine 2 | cholestasis | 1 |
| Cytarabine | cholestasis | 1 |
| Cytarabine 2 | cholestasis | 1 |
| Cytarabine 3 | cholestasis | 1 |
| Didanosine | cholestasis | 1 |
| Erythromycin | cholestasis | 1 |
| Ethosuximide | cholestasis | 0 |
| Flunitrazepam | cholestasis | 0 |
| Fluoxetine | cholestasis | 1 |
| Fluoxetine 2 | cholestasis | 1 |
| Fluoxetine 3 | cholestasis | 1 |
| Fluoxetine 4 | cholestasis | 1 |
| Haloperidol | cholestasis | 1 |
| Kanamycin | cholestasis | 0 |
| Levitra | cholestasis | 0 |
| Lovastatin | cholestasis | 0 |
| Netilmicin | cholestasis | 0 |
| Nevirapine | cholestasis | 1 |
| Omeprazole | cholestasis | 1 |
| Omeprazole 2 | cholestasis | 1 |
| Omeprazole 3 | cholestasis | 1 |
| Omeprazole 4 | cholestasis | 1 |
| Phenobarbital | cholestasis | 1 |
| Phenobarbital 2 | cholestasis | 1 |
| Phenytoin | cholestasis | 1 |
| Phenytoin 2 | cholestasis | 1 |
| Procaine | cholestasis | 0 |
| Propoxyphene | cholestasis | 1 |
| Propoxyphene 2 | cholestasis | 1 |
| Ritonavir | cholestasis | 1 |
| Rofecoxib | cholestasis | 1 |
| Rofecoxib 2 | cholestasis | 1 |
| Rofecoxib 3 | cholestasis | 1 |
| Stavudine | cholestasis | 1 |
| Stavudine 2 | cholestasis | 1 |
| Streptomycin | cholestasis | 0 |
| Sulfadoxine | cholestasis | 0 |
| Sulfamethoxazole | cholestasis | 1 |
| Sulfasalazine | cholestasis | 1 |
| Sulfasalazine 2 | cholestasis | 1 |
| Sulfasalazine 3 | cholestasis | 1 |
| Sulfasalazine 4 | cholestasis | 1 |
| Tadalafil | cholestasis | 0 |
| Tamoxifen | cholestasis | 1 |
| Tamoxifen 2 | cholestasis | 1 |
| Tamoxifen 3 | cholestasis | 1 |
| Tenoxicam | cholestasis | 0 |
| Ticlopidine | cholestasis | 1 |
| Tolbutamide | cholestasis | 1 |
| Trimethoprim | cholestasis | 1 |
| Valdecoxib | cholestasis | 0 |
| Valproate | cholestasis | 0 |
| Vardenafil | cholestasis | 0 |
| Zidovudine | cholestasis | 1 |
| Zidovudine 2 | cholestasis | 1 |
| Abacavir | deafness | 0 |
| Allopurinol | deafness | 0 |
| Amikacin | deafness | 1 |
| Amikacin 2 | deafness | 1 |
| Ampicillin | deafness | 0 |
| Aprindine | deafness | 0 |
| Bumetanide | deafness | 1 |
| Bumetanide 2 | deafness | 1 |
| Cephalexin | deafness | 0 |
| Cerivastatin | deafness | 0 |
| Chloramphenicol | deafness | 1 |
| Chloroquine | deafness | 1 |
| Chloroquine 2 | deafness | 1 |
| Chlorpromazine | deafness | 0 |
| Ciprofloxacin | deafness | 1 |
| Ciprofloxacin 2 | deafness | 1 |
| Cyproheptadine | deafness | 0 |
| Doxycycline | deafness | 1 |
| Doxycycline 2 | deafness | 1 |
| Erythromycin | deafness | 1 |
| Ethosuximide | deafness | 0 |
| Flunitrazepam | deafness | 0 |
| Furosemide | deafness | 1 |
| Furosemide 2 | deafness | 1 |
| Gentamicin | deafness | 1 |
| Gentamicin 2 | deafness | 1 |
| Gentamicin 3 | deafness | 1 |
| Haloperidol | deafness | 0 |
| Ibuprofen | deafness | 1 |
| Ibuprofen 2 | deafness | 1 |
| Ibuprofen 3 | deafness | 1 |
| Ibuprofen 4 | deafness | 1 |
| Indomethacin | deafness | 1 |
| Kanamycin | deafness | 1 |
| Kanamycin 2 | deafness | 1 |
| Kanamycin 3 | deafness | 1 |
| Kanamycin 4 | deafness | 1 |
| Levitra | deafness | 1 |
| Levofloxacin | deafness | 1 |
| Lovastatin | deafness | 0 |
| Metronidazole | deafness | 1 |
| Minocycline | deafness | 1 |
| Minocycline 2 | deafness | 1 |
| Netilmicin | deafness | 1 |
| Netilmicin 2 | deafness | 1 |
| Omeprazole | deafness | 0 |
| Phenobarbital | deafness | 0 |
| Phenytoin | deafness | 0 |
| Pravastatin | deafness | 0 |
| Procaine | deafness | 1 |
| Procaine 2 | deafness | 1 |
| Propoxyphene | deafness | 0 |
| Propranolol | deafness | 1 |
| Propranolol 2 | deafness | 1 |
| Quinine | deafness | 1 |
| Quinine 2 | deafness | 1 |
| Rofecoxib | deafness | 0 |
| Sildenafil | deafness | 1 |
| Sildenafil 2 | deafness | 1 |
| Simvastatin | deafness | 0 |
| Streptomycin | deafness | 1 |
| Streptomycin 2 | deafness | 1 |
| Sulfadoxine | deafness | 0 |
| Tadalafil | deafness | 1 |
| Tenoxicam | deafness | 0 |
| Tetracycline | deafness | 1 |
| Ticlopidine | deafness | 0 |
| Tobramycin | deafness | 1 |
| Tobramycin 2 | deafness | 1 |
| Tolbutamide | deafness | 0 |
| Valdecoxib | deafness | 0 |
| Vardenafil | deafness | 1 |
| Estradiol | estrogen receptor modulator | \N |
| Ethinyl estradiol | estrogen receptor modulator | \N |
| Fulvestrant | estrogen receptor modulator | \N |
| Genistein | estrogen receptor modulator | \N |
| Raloxifene | estrogen receptor modulator | \N |
| Tamoxifen | estrogen receptor modulator | \N |
| Hc toxin | HDAC inhibitor | \N |
| Trichostatin a | HDAC inhibitor | \N |
| Valproic acid | HDAC inhibitor | \N |
| Vorinostat | HDAC inhibitor | \N |
| 17-allylamino-17-demethoxygeldanamycin | HSP90 inhibitor | \N |
| Gedunin | HSP90 inhibitor | \N |
| Geldanamycin | HSP90 inhibitor | \N |
| Acetohexamide | longQT | 0 |
| Amiodarone | longQT | 1 |
| Amitryptiline | longQT | 1 |
| Amitryptiline 2 | longQT | 1 |
| Bethanidine | longQT | 0 |
| Bupivacaine | longQT | 1 |
| Celecoxib | longQT | 1 |
| Diazoxide | longQT | 0 |
| Diethylpropion | longQT | 0 |
| Disopyramide | longQT | 1 |
| Droperidol | longQT | 1 |
| Etoricoxib | longQT | 1 |
| Flecainide | longQT | 1 |
| Fluconazole | longQT | 1 |
| Fluoxetine | longQT | 1 |
| Fluoxetine 2 | longQT | 1 |
| Furosemide | longQT | 1 |
| Guanethidine | longQT | 0 |
| Haloperidol | longQT | 1 |
| Isocarboxazid | longQT | 0 |
| Loratadine | longQT | 1 |
| Lumiracoxib | longQT | 0 |
| Meloxicam | longQT | 0 |
| Meloxicam 2 | longQT | 0 |
| Mephenytoin | longQT | 0 |
| Oxprenolol | longQT | 0 |
| Phenylbutazone | longQT | 0 |
| Phenytoin | longQT | 0 |
| Pipobroman | longQT | 0 |
| Quetiapine | longQT | 1 |
| Quinidine | longQT | 1 |
| Rofecoxib | longQT | 1 |
| Rosiglitazone | longQT | 1 |
| Sibutramine | longQT | 1 |
| Sotalol | longQT | 1 |
| Sulfamethoxazole | longQT | 1 |
| Tacrine | longQT | 0 |
| Terfenadine | longQT | 1 |
| Tinidazole | longQT | 0 |
| Tridihexethyl | longQT | 0 |
| Trimethoprim | longQT | 1 |
| Triprolidine | longQT | 0 |
| 2,4-thiazolidinedione | obesity related drug | \N |
| 9-deoxy-delta-9-prostaglandin d2 | obesity related drug | \N |
| Indomethacin | obesity related drug | \N |
| Rosiglitazone | obesity related drug | \N |
| Rosiglitazone 2 | obesity related drug | \N |
| Troglitazone | obesity related drug | \N |
| Amphetamine | rhabdomyolysis | 1 |
| Amphetamine 2 | rhabdomyolysis | 1 |
| Amphetamine 3 | rhabdomyolysis | 1 |
| Amphetamine 4 | rhabdomyolysis | 1 |
| Aprindine | rhabdomyolysis | 0 |
| Atorvastatin | rhabdomyolysis | 1 |
| Atorvastatin 2 | rhabdomyolysis | 1 |
| Bumetanide | rhabdomyolysis | 1 |
| Bumetanide 2 | rhabdomyolysis | 1 |
| Cerivastatin | rhabdomyolysis | 1 |
| Chloramphenicol | rhabdomyolysis | 0 |
| Chloroquine | rhabdomyolysis | 0 |
| Chlorpromazine | rhabdomyolysis | 1 |
| Cyproheptadine | rhabdomyolysis | 0 |
| Cytarabine | rhabdomyolysis | 0 |
| Didanosine | rhabdomyolysis | 0 |
| Ethambutol | rhabdomyolysis | 0 |
| Ethosuximide | rhabdomyolysis | 0 |
| Fluvastatin | rhabdomyolysis | 1 |
| Furosemide | rhabdomyolysis | 1 |
| Furosemide 2 | rhabdomyolysis | 1 |
| Haloperidol | rhabdomyolysis | 1 |
| Isoflurane | rhabdomyolysis | 1 |
| Kanamycin | rhabdomyolysis | 0 |
| Levitra | rhabdomyolysis | 0 |
| Lovastatin | rhabdomyolysis | 1 |
| Lovastatin 2 | rhabdomyolysis | 1 |
| Methadone | rhabdomyolysis | 1 |
| Methadone 2 | rhabdomyolysis | 1 |
| Methadone 3 | rhabdomyolysis | 1 |
| Methadone 4 | rhabdomyolysis | 1 |
| Methadone 5 | rhabdomyolysis | 1 |
| Methadone 6 | rhabdomyolysis | 1 |
| Netilmicin | rhabdomyolysis | 0 |
| Nevirapine | rhabdomyolysis | 0 |
| Phenobarbital | rhabdomyolysis | 1 |
| Pravastatin | rhabdomyolysis | 1 |
| Pravastatin 2 | rhabdomyolysis | 1 |
| Procaine | rhabdomyolysis | 0 |
| Risperidone | rhabdomyolysis | 1 |
| Risperidone 2 | rhabdomyolysis | 1 |
| Risperidone 3 | rhabdomyolysis | 1 |
| Risperidone 4 | rhabdomyolysis | 1 |
| Simvastatin | rhabdomyolysis | 1 |
| Simvastatin 2 | rhabdomyolysis | 1 |
| Streptomycin | rhabdomyolysis | 0 |
| Succinylcholine | rhabdomyolysis | 1 |
| Succinylcholine 2 | rhabdomyolysis | 1 |
| Sulfadoxine | rhabdomyolysis | 0 |
| Sulfasalazine | rhabdomyolysis | 0 |
| Tadalafil | rhabdomyolysis | 0 |
| Tamoxifen | rhabdomyolysis | 0 |
| Tenoxicam | rhabdomyolysis | 0 |
| Terbutaline | rhabdomyolysis | 1 |
| Terbutaline 2 | rhabdomyolysis | 1 |
| Terbutaline 3 | rhabdomyolysis | 1 |
| Terbutaline 4 | rhabdomyolysis | 1 |
| Terbutaline 5 | rhabdomyolysis | 1 |
| Terbutaline 6 | rhabdomyolysis | 1 |
| Tolbutamide | rhabdomyolysis | 0 |
| Vardenafil | rhabdomyolysis | 0 |
| Abacavir | Stevens-Johnson Syndrome | 1 |
| Allopurinol | Stevens-Johnson Syndrome | 1 |
| Allopurinol 2 | Stevens-Johnson Syndrome | 1 |
| Amoxicillin | Stevens-Johnson Syndrome | 1 |
| Amoxicillin 2 | Stevens-Johnson Syndrome | 1 |
| Ampicillin | Stevens-Johnson Syndrome | 1 |
| Aprindine | Stevens-Johnson Syndrome | 0 |
| Atorvastatin | Stevens-Johnson Syndrome | 0 |
| Bumetanide | Stevens-Johnson Syndrome | 1 |
| Carbamazepine | Stevens-Johnson Syndrome | 1 |
| Carbamazepine 2 | Stevens-Johnson Syndrome | 1 |
| Celecoxib | Stevens-Johnson Syndrome | 1 |
| Cephalexin | Stevens-Johnson Syndrome | 1 |
| Chloramphenicol | Stevens-Johnson Syndrome | 0 |
| Chlorpromazine | Stevens-Johnson Syndrome | 0 |
| Ciprofloxacin | Stevens-Johnson Syndrome | 1 |
| Cyproheptadine | Stevens-Johnson Syndrome | 0 |
| Diclofenac | Stevens-Johnson Syndrome | 1 |
| Didanosine | Stevens-Johnson Syndrome | 1 |
| Didanosine 2 | Stevens-Johnson Syndrome | 1 |
| Didanosine 3 | Stevens-Johnson Syndrome | 1 |
| Doxycycline | Stevens-Johnson Syndrome | 1 |
| Doxycycline 2 | Stevens-Johnson Syndrome | 1 |
| Erythromycin | Stevens-Johnson Syndrome | 1 |
| Ethambutol | Stevens-Johnson Syndrome | 1 |
| Ethambutol 2 | Stevens-Johnson Syndrome | 1 |
| Ethosuximide | Stevens-Johnson Syndrome | 1 |
| Ethosuximide 2 | Stevens-Johnson Syndrome | 1 |
| Fluconazole | Stevens-Johnson Syndrome | 1 |
| Flunitrazepam | Stevens-Johnson Syndrome | 1 |
| Fluoxetine | Stevens-Johnson Syndrome | 1 |
| Fluoxetine 2 | Stevens-Johnson Syndrome | 1 |
| Fluoxetine 3 | Stevens-Johnson Syndrome | 1 |
| Fluoxetine 4 | Stevens-Johnson Syndrome | 1 |
| Haloperidol | Stevens-Johnson Syndrome | 0 |
| Ibuprofen | Stevens-Johnson Syndrome | 1 |
| Ibuprofen 2 | Stevens-Johnson Syndrome | 1 |
| Ibuprofen 3 | Stevens-Johnson Syndrome | 1 |
| Ibuprofen 4 | Stevens-Johnson Syndrome | 1 |
| Indomethacin | Stevens-Johnson Syndrome | 0 |
| Kanamycin | Stevens-Johnson Syndrome | 0 |
| Lamotrigine | Stevens-Johnson Syndrome | 1 |
| Lovastatin | Stevens-Johnson Syndrome | 0 |
| Metronidazole | Stevens-Johnson Syndrome | 0 |
| Minocycline | Stevens-Johnson Syndrome | 1 |
| Minocycline 2 | Stevens-Johnson Syndrome | 1 |
| Netilmicin | Stevens-Johnson Syndrome | 0 |
| Nevirapine | Stevens-Johnson Syndrome | 1 |
| Phenobarbital | Stevens-Johnson Syndrome | 1 |
| Phenobarbital 2 | Stevens-Johnson Syndrome | 1 |
| Phenytoin | Stevens-Johnson Syndrome | 1 |
| Phenytoin 2 | Stevens-Johnson Syndrome | 1 |
| Piroxicam | Stevens-Johnson Syndrome | 1 |
| Piroxicam 2 | Stevens-Johnson Syndrome | 1 |
| Pravastatin | Stevens-Johnson Syndrome | 0 |
| Procaine | Stevens-Johnson Syndrome | 0 |
| Propoxyphene | Stevens-Johnson Syndrome | 0 |
| Rofecoxib | Stevens-Johnson Syndrome | 1 |
| Rofecoxib 2 | Stevens-Johnson Syndrome | 1 |
| Rofecoxib 3 | Stevens-Johnson Syndrome | 1 |
| Simvastatin | Stevens-Johnson Syndrome | 0 |
| Sulfadoxine | Stevens-Johnson Syndrome | 1 |
| Sulfamethoxazole | Stevens-Johnson Syndrome | 1 |
| Sulfasalazine | Stevens-Johnson Syndrome | 1 |
| Sulfasalazine 2 | Stevens-Johnson Syndrome | 1 |
| Sulfasalazine 3 | Stevens-Johnson Syndrome | 1 |
| Sulfasalazine 4 | Stevens-Johnson Syndrome | 1 |
| Tadalafil | Stevens-Johnson Syndrome | 0 |
| Tenoxicam | Stevens-Johnson Syndrome | 1 |
| Tenoxicam 2 | Stevens-Johnson Syndrome | 1 |
| Tetracycline | Stevens-Johnson Syndrome | 0 |
| Ticlopidine | Stevens-Johnson Syndrome | 0 |
| Tolbutamide | Stevens-Johnson Syndrome | 1 |
| Valdecoxib | Stevens-Johnson Syndrome | 1 |
| Valdecoxib 2 | Stevens-Johnson Syndrome | 1 |
| Valproate | Stevens-Johnson Syndrome | 1 |

^#^ An entry name that is ended with a number represents a derivative of the corresponding parent drug, e.g., the isomers or its major metabolites.

* For a particular class, “1” or “0” means the drug was or was not found to cause this adverse drug reaction. “\N” denotes the drug is from the first paper describing cMAP.
