# Supplementary material for: Exploring Off-Targets and Off-Systems for Adverse Drug Reactions via Chemical-Protein Interactome — Clozapine-Induced Agranulocytosis as a Case Study
Source: PLoS Comput Biol. 2011 Mar 31;7(3):e1002016. doi: 10.1371/journal.pcbi.1002016 (PMC3068927; doi:10.1371/journal.pcbi.1002016)
Supplement: Table S3 — ANOVA of the chemical-protein interactive effect before and after 2-directional Z-transformation. (DOC) [file pcbi.1002016.s006.doc]

Table S3. ANOVA of the chemical-protein interactive effect before and after 2-directional Z-transformation

| Before 2DIZ | |  |  |  |
| --- | --- | --- | --- | --- |
|  | Df | Sum Sq | F | p value |
| Protein | 409 | 2332527 | 111.22 | <2.2e-16 |
| Chemical | 254 | 10330585 | 793.27 | <2.2e-16 |
| Interactive | 95344 | 4888387 |  |  |
| After 2DIZ | |  |  |  |
| Protein | 409 | 0 | 1.37E-19 | 1 |
| Chemical | 254 | 1052 | 4.1776 | <2.2e-16 |
| Interactive | 95344 | 94546 |  |  |
